# A Mononuclear Scenario for the Copper‐Catalyzed Monooxygenation of Phenolic Substrates

**DOI:** 10.1002/chem.202503505

**Published:** 2026-03-24

**Authors:** Alexander Koch, Antony Memboeuf, Felix Tuczek, Tobias A. Engesser

**Affiliations:** ^1^ Institut Für Anorganische Chemie Christian‐Albrechts‐Universität Zu Kiel Kiel Germany; ^2^ Univ. Brest CNRS, UMR 6521 CEMCA Brest France

**Keywords:** copper catalysis, mass spectrometry, oxygenation, reaction mechanism, tyrosinase

## Abstract

Whereas a dinuclear pathway applies to the monooxygenation of phenols by the type 3 copper enzyme tyrosinase, a mononuclear pathway has been identified for the same reaction in corresponding small‐molecule systems. DFT calculations of five copper model complexes reveal that the latter mechanism is energetically feasible, identifying all intermediates, and transition states. The mechanism closely resembles the biosynthesis of the topaquinone (TPQ) cofactor in the enzyme amine oxidase (AO), though the latter process is not catalytic. Additionally, it is shown that the hydroxo‐quinone complex formed after the hydroxylation step further converts into a copper‐geminal diolate species, which is confirmed by MS/MS experiments using isotope labeling with ^18^O_2_.

## Graphical abstract

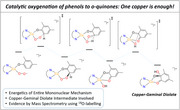

## 1 Introduction

Type 3 copper proteins are a class of enzymes that contain a dinuclear copper center as the active site, with each copper ion coordinated by three histidine ligands [1]. This group includes tyrosinase (Ty), catechol oxidase (CO), and hemocyanin (HC). Among them, Ty plays a crucial role in oxygenation reactions, catalyzing both the hydroxylation of monophenols to *o*‐diphenols and the subsequent oxidation of *o*‐diphenols to *o*‐quinones, in nature realized by the conversion of tyrosine to dopaquinone [2, 3, 4, 5, 6]. This enzymatic activity is fundamental in processes such as melanin biosynthesis, enzymatic browning [7, 8, 9], wound healing and immune response [10, 11, 12].

**SCHEME 1:**
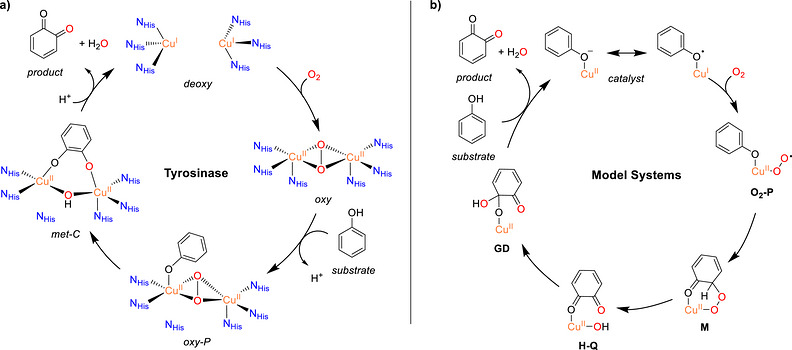
(a) The dinuclear catalytic cycle of tyrosinase and (b) the proposed mononuclear cycle for model systems. **O_2_‐P**, dioxygen phenolate complex; **M**, metallacycle; **H‐Q**, hydroxo‐quinone species; **GD**, geminal diolate. The N donor coligands are omitted for clarity.

**SCHEME 2:**
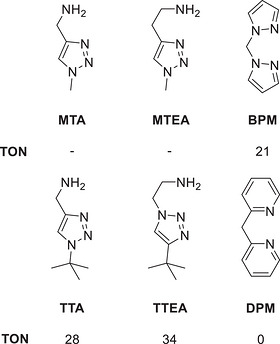
The symmetric **BPM**, **DPM**, and asymmetric **TTA**, **MTA**, and **MTEA** ligands investigated in this study; the latter two were derived from **TTA** and **TTEA**, respectively. Turnover numbers (TON) for the conversion of **DTBP‐H** to **DTBQ** [23, 24, 26].

Important structural information on Ty has been obtained through crystallographic studies on the isolated enzyme of Streptomyces castaneoglobisporus, which revealed a side‐on bridging (μ‐η^2^:η^2^) peroxo dicopper(II) core as the active species responsible for oxygenation reactions (Scheme 1a) [13, 14]. To further understand the activity of type 3 copper enzymes and the underlying molecular mechanism as well as to design efficient catalysts for the mediated oxidation reactions, artificial model systems mimicking the reactivity have been developed (for reviews see [15, 16, 17, 18, 19, 20]). Accordingly, the first Ty‐like reactivity and conversion of monophenols to quinones was achieved with catalysts containing dinucleating ligands copying the structure of the enzyme. Early examples are the dinuclear copper(I) complexes [Cu_2_(MeCN)_4_ **BiPh(impy)_2_**](PF_6_)_2_ and [Cu_2_(**L‐66**)] reported by Réglier [21] and casella, respectively [22]. More recently, catalysts containing mononucleating ligands were presented by our group, starting with a pyridylethylimine ligand (**L_py_^1^**) in 2010 [23], followed by bis(pyrazolyl)methane (**BPM**) [24] and several (triazolylmethyl)pyridine‐ (**TMP**) [25], aminomethyltriazole‐ (**TTA**) as well as aminoethyltriazole‐based systems (**TTEA**) for instance (Scheme 2) [26]. Interestingly, there are also complexes with very similar ligands, such as dipyridylmethane (**DPM**), which have been found to be inactive under the same conditions, which underlines the importance of a well‐chosen ligand design.

For many years, the enzyme's dinuclear reaction mechanism was assumed to also be the basis for the reactivity of the corresponding model systems [23, 27, 28, 29, 30, 31]. This was also based on detailed theoretical investigations, for example with DFT by Siegbahn et al. [32, 33] or with QM/MM by Yoshizawa et al. [34]. Surprisingly, however, we could lately present compelling evidence that some of the Ty‐like oxygenation reactions mediated by model systems can actually also follow a mononuclear mechanistic pathway [35, 36]. It was proposed that this alternative mechanism involves a copper(II)‐phenolate or copper(I)‐phenoxyl species (Scheme 1b, *catalyst*). Binding of dioxygen most likely forms an end‐on dioxygen intermediate (Scheme 1b, **O_2_‐P**), enabling electrophilic attack at the ortho position of the phenol substrate to form a copper metallacycle (Scheme 1b, **M**). Subsequent O─O bond cleavage leads to the formation of a hydroxo‐quinone species (Scheme 1b, **H‐Q**), which, as we will show herein, may further react to a geminal diolate (Scheme 1b, **GD**). Upon addition of the next substrate molecule, the quinone product is released and the catalyst is regenerated.

Despite experimental evidence from kinetic investigations suggesting that the catalytic conversion proceeds via a mononuclear pathway, key mechanistic intermediates, such as the proposed six‐ring metallacycle (**M**) or the hydroxo‐quinone (**H‐Q**) species remained spectroscopically elusive, due to their high instability or short lifetimes. This renders it necessary to study the mechanism computationally. A mechanism involving only one copper center, which is presented herein, offers several practical advantages with respect to that involving a dinuclear copper system, including reduced computational cost and greater tractability, not to mention the fact that the latter tends to have multireference character and convergence problems. Thus, all relevant intermediates and transition states of several different ligands in solution can be modelled with high accuracy. This potentially allows a detailed exploration of the entire reaction sequence, validating the proposed mechanism by assessing the energetic feasibility of each step and identifying the rate‐determining states. Additionally, hidden or transient intermediates may be revealed and the question of how structural features influence reactivity may be answered. Our goal is, therefore, not only to support the mononuclear Ty‐like mechanism, but also to establish structure–reactivity relationships that go beyond the simple interpretation of activity by TONs and TOFs, which was done before [37]. Moreover, the obtained information can also help to design experiments to prove specific theoretical predictions relating to, for example, the structure of key intermediates. Notably, the closely related mechanism of dinuclear copper systems has already been the subject of several previous studies, so comparison with these results is, of course, of great interest as well, particularly with regard to elucidating the role of the second copper in the enzyme.

Herein, we present a comprehensive theoretical analysis of five mononuclear copper model systems catalyzing the monooxygenation of phenols, focusing on key aspects that influence their performance. We describe the modeling of the catalysts and substrate, detailing their structural, and electronic properties. This is followed by the discussion of the thermodynamics of the mechanism, evaluating the energetics of key reaction steps to understand the driving forces behind the process, and the identification of crucial intermediates and transition states. Finally, we present experimental evidence of a copper‐geminal diolate intermediate by mass spectrometry, which plays an important role in the presented catalytic cycle and has not been described yet.

## 2 Results and Discussion

In our previous experimental studies, we observed that the catalytic Ty‐like activity of copper complexes with bidentate N donor ligands depends on several factors, including the type of donor (e.g., imidazole [38], benzimidazole [39], triazole [25, 26, 35], pyrazole [24, 40], amine [26], or imine [23, 35]), ligand symmetry (symmetric bis‐N donor systems **BPM** or **BIMZ** vs. asymmetric mixed N donor systems), and ligand flexibility (e.g., length of alkyl chain) [26]. Based on these findings, we selected five systems for this study that incorporate as many different parameters as possible while ensuring they remain comparable (Scheme 2).

Currently, the most active catalysts feature triazole donors, such as **TMP** [25], **L**_**trz**_ **1/2** [35], or **TTA** [26]. Therefore, we used one of these (**TTA**) for our study. To investigate the impact of the chain length on the catalytic activity, two asymmetric systems (**MTA** and **MTEA**) were designed, which only differ in their chain length and which also include triazoles as one of the N donors (Scheme 2). To make the design as simple as possible and to save computation time, the triazoles in these two systems bear a methyl group instead of the *tert*‐butyl group of the analogues **TTA** and **TTEA**. Furthermore, the triazoles in the model ligands carry simple aminoalkyl substituents like in the experimentally investigated systems. It should be mentioned that in **TTA** and **TTEA**, the tert‐butyl residues are in different positions of the triazole moiety (Scheme 2), due to their different syntheses. For the present study, we decided to design the best comparable model systems possible, so **MTA** and **MTEA** have both the methyl group in 1‐position, which means that with the chain length of the alkylamine group only one parameter is changed instead of two; however, **MTEA** is not exactly the methyl‐analogue of **TTEA**. **BPM** was chosen as a symmetric, ligand giving rise to a catalytically active system whereas **DPM** was included to investigate whether the inactivity of the derived copper complex would be reflected in the energetics of the mechanism and thus could be better understood. In summary, we decided to use copper complexes (**CuL**) supported by the mentioned bidentate N donor ligands for the calculations, including asymmetric (*L* = **TTA**, **MTA**, and **MTEA**) and symmetric ligands (**BPM** and **DPM**) [24] as examples for highly active, inactive and well‐comparable systems, respectively, with the goal to understand the differences in their activities (Scheme 2).

**FIGURE 1:**
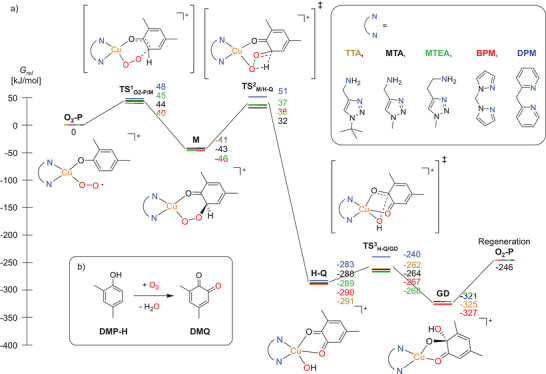
(a) Calculated energy profile for copper‐catalyzed oxidation of dimethylphenol (**DMP‐H**) to dimethylquinone (**DMQ**) by catalysts **CuBPM**, **CuDPM**, **CuTTA**, **CuMTA**, and **CuMTEA** in solution (DCM, *ε* = 8.93, PBE0‐D3(BJ)/def2‐TZVPP). **O_2_‐P** = dioxygen phenolate complex, **M** = metallacycle, **H‐Q** = hydroxo quinone complex, **GD** = geminal diolate complex. Relative free energies *G_rel_* [kJ·mol^−1^] are given with respect to the starting complex (**O_2_‐P**). Regeneration is accompanied by addition of O_2_, substrate (**DMP‐H**) and the formation of H_2_O and product (**DMQ**). (b) Model reaction of the phenolic model substrate 2,4‐**DMP‐H** to *ortho‐*quinone 3,5‐**DMQ**. For structures of the complexes see Figures 2 and S4–S11.

The chosen model substrate 2,4‐dimethylphenol (**dmp‐h**; Figure 1) has similar electronic properties as the substrate 2,4‐di‐tert‐butylphenol (**DTBP‐H**), which has been used in many experimental studies, including **CuBPM** [24], **CuTMP** [25], as well as **CuTTA**, and **CuTTEA** [26] as catalysts. For the present calculations, these complexes are chosen either directly (**BPM**, **DPM, TTA**) or with truncated ligands (**MTA**, **MTEA**). Replacement of the *tert*‐butyl by methyl groups should have little influence on the electronic properties as the residues face outward and are not close to the copper center, the influence of the different steric effects on the reactivity should be negligible as well. This can also be examined by comparison of **TTA** and **MTA**. Based on our previous findings that weakly coordinating anions (WCAs) can enhance the catalytic activity [36], all species were modeled as cationic, partially coordinatively unsaturated complexes, and the anion was omitted from the computational study. Explicit inclusion of anions is not expected to significantly affect the energetics due to only weak axial interactions, while substantially increasing computational cost and rendering transition‐state searches impractical.

The calculations were performed in solution (DCM, *ε* = 8.93) at the PBE0‐D3(BJ)/def2‐TZVPP level. PBE0 was chosen as there seem to be small differences between the accuracy of different functionals, for instance for first row transition metal systems [41] and compared to the more modern QM/MM hybrid methods and especially for large systems, DFT is still able to provide accurate results, and at a relatively low computational cost [42] (for details to the method see Supporting Information and Figure S1). With a calculated free reaction enthalpy of approximately −246 kJ·mol^−1^, the reaction of the model substrate **DMP‐H** to 2,4‐dimethylquinone (**DMQ**) and water (Figure 1) is highly exergonic. Consequently, each catalytic cycle releases this amount of energy per turnover.

### 2.1 Mononuclear Scenario for the Monooxygenation of Phenolic Substrates

**FIGURE 2:**
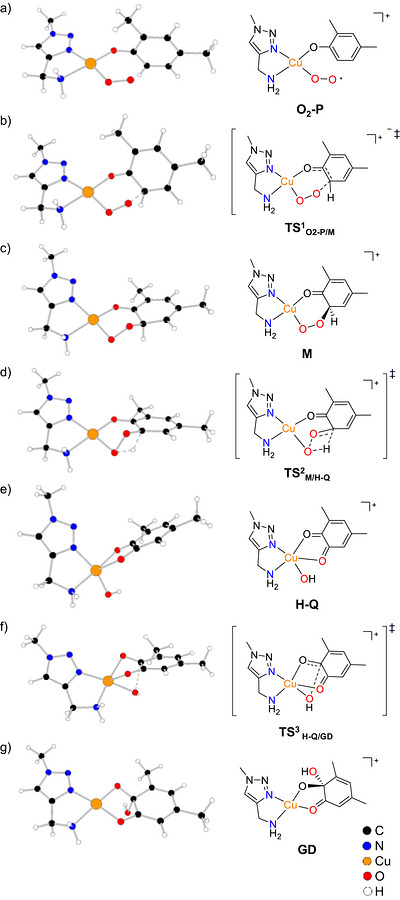
Intermediates and transition states of the mononuclear pathway of the **CuMTA** system including (a) dioxygen phenolate complex (**O_2_‐P**) (b) the first transition state **TS^1^_O2‐P/M_**, (c) metallacycle (**M**) (d) the second transition state **TS^2^_M/H‐Q_**, (e) hydroxo complex (**H‐Q**), the third transition state **TS^3^_H‐Q/GD_**, and (g) geminal diolate complex (**GD**).

The catalytic oxidation of the model substrate **DMP‐H** to **DMQ** proceeds via several steps, and for the calculation of the entire process it is crucial to determine the optimal entry point or starting complex. We decided to initiate the calculated reaction sequence with the dioxygen complex (Figures 1 and 2a, **O**_**2**_ **‐P**). First, the energetics of its formation is difficult to model, and secondly, it represents a reasonable starting point since copper(I) complexes are known to form dioxygen or superoxo complexes in the presence of O_2_ [43, 44, 45]. Furthermore, similar mechanisms of copper enzymes with mononuclear active sites start with superoxo complexes (e.g., dopamine‐β‐monooxygenase (DbH) [46] and peptidlyglycine‐α‐hydroxylating monooxygenase (PHM) [47], amine oxidase (AO) [48], or the biogenesis of TPQ in AO [49]) whereas dinuclear copper systems form μ‐η^2^:η^2^‐peroxo or trans‐μ‐1,2‐peroxo dicopper(II) cores [50]. Finally, if the reactive cycle starts with a dioxygen complex, it is reformed in the final step, which includes the addition of the next substrate molecule (**DMP‐H**) and O_2_, accompanied by the release of the product (**DMQ**).

As with all presented ligands several conformers are possible, the entire cycle was calculated at least for two of them. For the asymmetric ligands, for instance, the triazole or the amine donor can coordinate trans to the dioxygen ligand in **O**_**2**_ **‐** **P**. For **MTA** and **MTEA** the energetics for both are determined (Tables S1 and S2). For the symmetric ligands **BPM** and **DPM** the central CH_2_ group can point upward or downward with respect to the coordinating phenolate (Figure S3), and the energetics for both conformers were determined (Tables S3 and S4). Regarding the different conformers, the most energetically favorable structures and the corresponding scenario will be discussed (Figures 1 and 2, for more details about the conformational analysis, see Figures S2 and S3).

The coordination to copper increases the electrophilicity of the β‐oxygen atom of the dioxygen ligand in **O**_**2**_‐**P**, which can now react with the phenolate ligand in an intramolecular electrophilic aromatic attack (Figure 1, **O**_**2**_ **‐** **P** to **M**). The corresponding transition state already shows the six‐membered cupric metallacycle (Figures 1 and 2b, **TS^1^_O2‐P/M_**, for animation see Supporting Information) of the formed complex **M** (Figures 1 and 2c)), and the energy barrier with 40–48 kJ·mol^−1^ is compatible with a thermally allowed reaction (Tables S1–S5). Notably, it is highest in case of catalytically inactive **CuDPM** (48 kJ·mol^−1^). **M** resembles the typical sigma complexes of electrophilic aromatic substitution reactions, its formation being exergonic by −41 to −46 kJ·mol^−1^ (Tables S1–S5). Again, the **M** intermediate of the inactive **CuDPM** system is, together with **CuTTA,** with −41 kJ·mol^−1^ the least favorable of the five products and therefore has the lowest driving force to be formed. It should be mentioned that we already showed by an inverse kinetic isotope effect that the formation of the *σ*‐complex (here **M**) by attack of the electrophilic CuO_2_ species (here **O_2_‐P**) and not the C─H cleavage represents the rate‐limiting step [35, 36]. As in the presented cycle for the catalytically active systems (**CuTTA** and **CuBPM**), the corresponding transition state **TS^1^_O2‐P/M_** can be viewed as rate‐determining state [51], this is now further confirmed and fits to the experiment.

In the next step, the hydrogen at the sp^3^ carbon atom of the attacked substrate in **M** shifts to the copper‐bond O atom, which is the former α‐oxygen of the dioxygen ligand, via a four‐membered transition state (**TS^2^_M/H‐Q_**, Figures 1 and 2d, for animation see Supporting Information). The so‐formed hydroxo quinone complex [Cu(OH)(**DMQ**)L] (**H‐Q**, Figures 1 and 2e; *L* = bidentate coligand) now contains the quinone product (**DMQ**) as ligand. The energy barrier is with 75 kJ·mol^−1^ lowest in case of **CuMTA** and with 77 to 83 kJ·mol^−1^ a bit higher for **CuTTA**, **CuMTEA** and **Cu** **B** **PM**. Interestingly, for inactive **CuDPM** again the least favorable process is found with a significantly higher lying **TS^2^_M/H‐Q_** (92 kJ·mol^−1^), as it was the case for **TS^1^_O2‐P/M_** and **M**. The reactions from **M** to **H‐Q** are all similarly exergonic with −242 to −250 kJ·mol^−1^, and relative to the starting complex **O_2_‐P** the inactive **CuDPM** system is the least stable with −283 kJ·mol^−1^.

**H‐Q** now contains the quinone product which could be released under addition of O_2_ and formation of water from the hydroxo ligand, starting the next cycle. However, the calculations indicate that the nucleophilic hydroxo ligand at the copper center can also be transferred to the quinone moiety. More precisely, it can attack the electrophilic former phenolic (ipso) carbon, which has become sp^2^‐hybridized, forming a geminal diolate (**GD**, Figures 1 and 2g). The reaction proceeds via **TS^3^_H‐Q/GD_**, which clearly shows the transfer of the hydroxo ligand (Figures 1 and 2f, for animation see Supporting Information). Interestingly, again for **CuDPM** a less favorable transition state is found with −240 kJ·mol^−1^. It should be mentioned that the other possible conformer has a **TS^3^_H‐Q/GD_** which is energetically more similar to the other systems with −265 kJ·mol^−1^ (Table S4), but here **H‐Q** is much less stable with −268 kJ·mol^−1^. So, in any case **CuDPM** seems to have a less favorable pathway.

Regarding the location of the unpaired electron during the process, spin densities were calculated for the entire mechanism with **CuMTA** as catalyst (Figure S12). In case of the starting complex **O_2_‐P**, it is clearly visible that the compound is best described as copper(II) complex with a dioxygen and a phenolate ligand. Copper and dioxygen ligand seem to be antiferromagnetically coupled (open‐shell doublet) with the largest spin density on the β‐oxygen atom. The unpaired electron is mostly located in the one π*(O_2_) orbital, more specifically, the one which is not involved in the sigma bonding to the copper center. After the electrophilic aromatic attack and formation of **M**, the unpaired electron is mostly located on the copper center during the entire mechanism.

**SCHEME 3:**
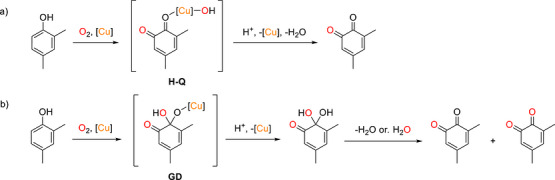
The origin of the oxygen atoms in the product, depending on different intermediates in the reaction mechanism, when (a) a hydroxo intermediate (**H‐Q**) or (b) a geminal diolate (**GD**) is the last intermediate before product release.

**SCHEME 4:**
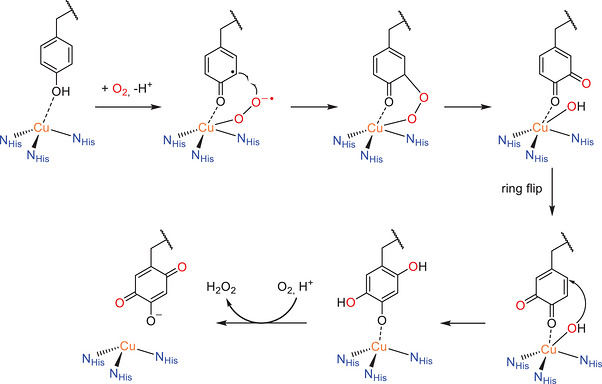
Simplified proposed mechanism for the biogenesis of the TPQ cofactor, including a transfer of the hydroxo ligand to the substrate (adapted from [52, 53]).

**GD**, a geminal diolate, has not been reported in Ty‐like mechanisms before, but the calculations clearly show that it is 31–41 kJ·mol^−1^ more stable than **H‐Q**. Furthermore, its involvement in the process is indeed not unlikely, as its protonation would lead to a geminal diol, which could potentially eliminate H_2_O under formation of a ketone, in this case the quinone product (**DMQ**; Scheme 3b). This suggests that **GD** represents the final intermediate before regeneration of the dioxygen complex in a mononuclear mechanism. Notably, in the mononuclear copper‐mediated mechanism of the biogenesis of the topaquinone (TPQ) cofactor one of the last steps also includes the nucleophilic attack of a copper‐bound hydroxo ligand to a sp^2^ ring carbon atom. In this case, however, the *meta* position to the phenolic carbon is hydroxylated and not the phenolic (ipso) carbon itself (Scheme 4) [52, 53].

Comparison of the calculated reaction profiles for the different ligand systems clearly reveals that **CuDPM**, the catalytically inactive complex, consistently exhibits the highest relative energies for all intermediates and transition states throughout the entire catalytic cycle. This indicates that the **DPM** ligand enforces the most unfavorable reaction pathway, with higher barriers and less stable intermediates, correlating well with its experimentally observed inactivity. Nevertheless, the energetic differences are not significant enough to explain a complete lack of activity. Furthermore, analyzing the structural differences between the intermediates and transition states also don't serve a clear explanation, such as strongly differing steric hinderance or bond strengths (for comparison of the parameters of **TS^2^_M/H‐Q_** and **TS^3^_H‐Q/GD_**, see Figure S13). Therefore, there has to be another reason, which most likely is an entirely different chemical behavior of **DPM**. Specifically, the C─H acidity of the methylene bridge in **DPM** is much higher than in the other three systems [54], therefore, it could get deprotonated under the basic catalytic conditions (excess NEt_3_). This would lead to an anionic ligand which in turn would be counterproductive with regard to the inner‐sphere hydroxylation of phenolate involved in the derived mechanistic cycle.

Going back to the energy profile, the other two catalytically active systems **CuTTA**, and **CuBPM** as well as the analogous systems **CuMTA** and **CuMTEA**, show remarkably similar energy profiles, suggesting comparable mechanistic behavior. A comparison of **CuMTA** and **CuMTEA** indicates that variations in chain length have little influence on the energetics. **CuBPM** most often displays the lowest‐energy transition states and intermediates, indicating a slightly more favorable reaction pathway. However, the energetic differences between the active systems are relatively small typically below 10 kJ·mol^−^^1^ implying that all three follow closely related and energetically accessible pathways, with the **CuDPM** system standing out as distinctly less favorable.

The regeneration of the dioxygen complex **O_2_‐P** from **GD**, via addition of O_2_ and the phenolic substrate **DMP‐H**, is calculated to be endergonic by 75–81 kJ·mol^−^^1^. At first glance, this appears problematic, as efficient regeneration of the catalyst is crucial for a productive catalytic cycle. However, the overall transformation remains highly exergonic, with a total free energy change of –246 kJ·mol^−^^1^ across the entire sequence. This substantial driving force, particularly the strongly exergonic step from **M** to **H‐Q** (Δ*G* = –242 to –250 kJ·mol^−^^1^), likely compensates for the thermodynamically uphill final step, enabling the catalytic cycle to proceed. Furthermore, it is important to note that the last step includes the formation of water, whose thermodynamic contribution can vary significantly depending on the solvent environment (here DCM). It is also conceivable that water is not formed as free, solvated H_2_O but rather remains coordinated or reacts further to form a yet unidentified species. Such uncertainties in the final state of water could affect the computed energetics. Taken together, although the final regeneration step appears endergonic in isolation, the full catalytic cycle remains viable due to the overall exergonic profile and possible solvent‐ or coordination‐dependent effects in the final transformation. It should be mentioned that it is still also possible that the hydroxo complex (**H‐Q**) gets directly protonated, water is formed and the coordinated quinone gets substituted by the next substrate molecule. This would be more in line with the classical dinuclear Ty mechanism and would make the regeneration endergonic by only 37–45 kJ·mol^−^^1^. Notably, the formation of **GD** involves a new C–O bond, opening the possibility to experimentally check whether this intermediate is in fact part of the catalytic cycle. This can be achieved through isotopic labeling in combination with spectroscopy, as described in the following.

### 2.2 Evidence for the Geminal Diolate Intermediate

In principle, a mechanism involving either **H‐Q** or **GD** as the final step can lead to quinone formation. This means that there could be a competition between a process which directly leads to the release of quinone from **H‐Q** or its reaction to the more stable **GD** via low lying transition state **TS^3^_H‐Q/GD_**. To experimentally verify the existence of this geminal diolate intermediate, we decided to examine the identity of the formed quinone with respect to the contained oxygen atoms. Notably, in the mechanism ending with **H‐Q** as last intermediate, only one of the oxygen atoms from O_2_ is incorporated in the product (Scheme 3a), which means that only one C─O bond is formed. In contrast, the mechanism ending with the geminal diolate (**GD**), should produce a mixture of two products, one of which forms through substitution of the original phenolic oxygen with an oxygen atom from O_2_ (Scheme 3b). Thus, by using ^18^O_2_​ as a reagent, it should be possible to check whether **GD** is involved in the mechanism. Specifically, if some of the quinone product contains two incorporated ^18^O atoms (**DTBQ**‐^18^O_2_), the mechanism involving **GD** as intermediate is the only plausible explanation. Even if only small amounts of **DTBQ**‐^18^O_2_ are found, the geminal diolate must have been involved in the process.

To examine the fate of the oxygen atoms during the catalytic processs, the conversion of 2,4‐di‐*tert*‐butylphenol (**DTBP**) into 3,5‐di‐*tert*‐butyl‐*ortho*‐quinone (**DTBQ**) was investigated under ^18^O_2_ atmosphere. As catalyst [Cu(**TTEA**)(NCMe)]PF_6_ was chosen, whose activity was previously investigated [26], and which is analogous to **CuMTEA** used for the calculations in this study. As before, a 500 µm solution of the copper(I) complex with 50 eq. of phenol and 100 eq. of triethylamine were employed, the so‐called Bulkowski–Réglier conditions [21, 55]. An NMR spectrum (Figure S14) indicated the succesful formation of **DTBQ** with a yield of 33%. The reaction product was then further investigated via mass spectrometry to determine the content of ^18^O in the product.

The obtained mass spectrum (Figure S15c) shows the formation of **DTBQ** (*m/z* 221), **DTBQ**‐^18^O (*m/z* 223), and **DTBQ**‐^18^O_2_ (*m/z* 225). The observation of a peak for **DTBQ** without ^18^O was unexpected under ^18^O_2_ atmosphere. It is possible that an oxygen exchange through reaction with water (H_2_^16^O) occurs due to traces present in the used dichloromethane, converting **DTBQ**‐^18^O and **DTBQ**‐^18^O_2_ to **DTBQ** after the catalytic reaction. It has to be further noted that the peaks assigned to **DTBQ** species are generally found to be exceptionally weak, which ruled out investigation via GC‐MS.

**FIGURE 3:**
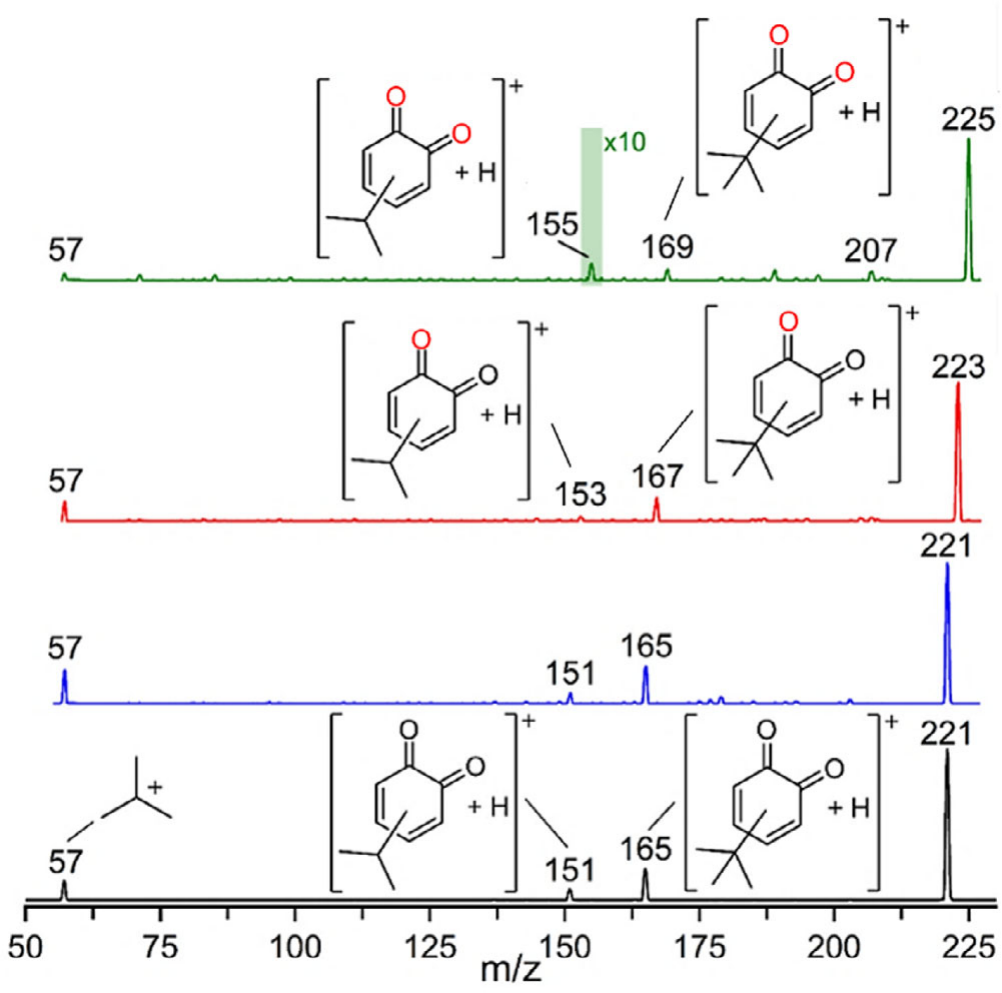
ESI‐MS/MS spectra on the [M+H]^+^ signals for commercially obtained **DTBQ** (black, 5 eV collision energy), **DTBQ** from the catalytic experiment (blue, 8 eV collision energy), **DTBQ**‐^18^O (red, 8 eV collision energy), and **DTBQ**‐^18^O_2_ (green, 8 eV collision energy). Red oxygen atoms represent ^18^O‐labeled atoms.

To demonstrate that the peaks with *m/z* 221, 233, and 225 are indeed unambigously linked to **DTBQ** species, MS/MS measurements were performed. At first, to prove the identity of the product and to understand the fragmentation products, commercially purchased **DTBQ** was measured (Figure S16). The ions at *m/z* 221 were then isolated and collided with Ar gas at a collision energy of 8 eV. In the corresponding mass spectrum (Figure 3), three fragment ions were observed at *m/z* 165, *m/z* 151, and *m/z* 57. The fragment at *m/z* 165 can be assigned to *tert*‐butyl‐*ortho*‐quinone, which is formed after homolytic C─C bond cleavage between the quinone ring and one *tert‐*butyl group leading to the loss of isobutylene (Scheme S1). The corresponding peak for protonated isobuytlene/ the *tert‐butyl* cation can be observed at *m/z* 57. The loss of isobutylene from *tert*‐butyl groups bound to a ring has been previously observed in ESI‐MS/MS spectra by Bouchoux et al. for the fragmentation of phenoxide ions [56]. In a similar fashion, an additional loss of CH_2_ from the remaining *tert*‐butyl group results in the formation of isopropylquinone (*m/z* 151, Figure 3 and Scheme S2).

In the next step, MS/MS experiments were now performed for the signals of **DTBQ** (*m/z* 221), **DTBQ**‐^18^O (*m/z* 223), and **DTBQ**‐^18^O_2_ (*m/z* 225) obtained from catalytic reactions with ^18^O_2_. The MS/MS spectrum on the *m/z* 221 peak from the catalytic run resembles the one obtained for pure, commercially obtained **DTBQ** (Figure 3). Moreover, the fragment ion peak at *m/z* 165 now shifts to *m/z* 167 and *m/z* 169 and the one at *m/z* 151 to *m/z* 153, and *m/z* 155, respectively (Figure 3), which proves that these peaks are ^18^O sensitive. The natural abundance of ^18^O is 0.2%, so it is clear that the signal of **DTBQ**‐^18^O_2_, which is almost as intense as the one for **DTBQ**‐^18^O, cannot orginate just from natural ^18^O‐**DTBP** (Figure S15). These results thus prove the formation of **DTBQ**‐^18^O_2_ and, therefore, the existence of a mechanistic pathway involving the geminal diolate intermediate (**GD**).

### 2.3 Comparison With Tyrosinase and Amine Oxidase

After showing that a mononuclear mechanism for Ty activity is energetically feasible, the question arises as to why in Ty a second copper is needed whereas, in the model systems, the same reactivity seems to be possible with only one copper center. An obvious answer to this question is the fact that, in nature, Ty also has to transform dopa, a catechol, into dopaquinone, which then reacts to dopachrome. The underlying synchronous two‐electron oxidation is only possible with two copper centers. Beyond this explanation, however, it is of interest to evaluate in which respect the presence of a second copper center affects the energetics of the monooxygenation pathway. Notably, Ty, forms bridging complexes, such as the μ‐η^2^:η^2^‐peroxo or μ‐hydroxo complexes, which of course strongly affects the energetics of the process. Therefore, further studies have to show whether the vicinity of a second copper in the model systems stabilizes the hydroxo quinone intermediate **H‐Q** and destabilizes the following transition state **TS^3^_H‐Q/GD_** or favors the direct formation of water, for instance. This would result in the formation of the quinone product directly from **H‐Q** and indicate that no intermediate such as the presented **GD** exists in the natural enzymatic process, implying that the latter is an exclusive feature of the reaction catalyzed by the mononuclear model systems. Alternatively, a **GD** intermediate also exists in the Ty mechanism, but was not found or described yet. Even if this is unlikely, due to the role of the second copper, the same isotope experiments with ^18^O_2_ as employed in this study could shed light on this.

Finally, as the mechanistic pathway presented herein exclusively involves mononuclear Cu(II) intermediates, it corresponds more to the (stoichiometric) TPQ cofactor synthesis in AO than to Ty. AO, whose primary role is the oxidation of amines to aldehydes under generation of H_2_O_2_, also post‐translationally converts a nearby tyrosine stoichiometrically to the TPQ cofactor, which is necessary for its function. The underlying mechanism strongly resembles the one presented here (Scheme 3) [57, 58, 59, 60]. Therefore, employing biomimetic small‐molecule copper model chemistry, we found a scenario strongly related to the stoichiometric process of AO, but for the catalytic conversion of external monophenolic substrates to *ortho*‐quinones.

## 3 Conclusion

In this study a mononuclear Ty‐like oxidation mechanism was investigated by using the copper complexes **CuTTA**, **CuMTA**, **CuMTEA, CuBPM**, and **CuDPM** as model systems. Two of these five examples have previously found to be potent catalysts, two are analogues for analyzing specific parameters (**CuMTA** and **CuMTEA**) and one, namely **CuDPM**, was chosen as example for an inactive system for comparison. The mononuclear scenario, which was described theoretically, represents a viable alternative to the classical dinuclear pathway observed in natural enzyme Ty. Through density functional theory (DFT) calculations, the energetic profiles with all intermediates and transition states were evaluated, revealing a mechanistically plausible sequence of substrate coordination, O_2_ activation, and subsequent hydroxylation. A distinctive feature of the proposed cycle is the formation of a geminal diolate (**GD**) intermediate, which was not described before. The existence of this intermediate was mass spectrometrically proven by isotopic labeling under ^1^ ^8^O_2_, confirming the incorporation of oxygen atoms derived from molecular oxygen. Furthermore, the computational data highlight how variations in the ligand environment influence activation barriers and intermediate stability, establishing clear structure–reactivity relationships. These findings deepen our understanding of copper‐mediated oxygenation processes and provide a foundation for future studies aimed at validating and optimizing copper‐based monooxygenation catalysts.

## Conflicts of Interest

The authors declare no conflicts of interest.

## Supporting information




**Supporting File 1**: chem70898‐sup‐0001‐SuppMat.pdf.

**Supporting File 2**: chem70898‐sup‐0002‐SuppMat.zip.
