# Supplementary material for: A Mononuclear Scenario for the Copper‐Catalyzed Monooxygenation of Phenolic Substrates
Source: Chemistry. 2026 Mar 24;32(27):e03505. doi: 10.1002/chem.202503505 (PMC13380385; doi:10.1002/chem.202503505)
Supplement: Supplementary file 1 — Supporting File 1: chem70898‐sup‐0001‐SuppMat.pdf. [file CHEM-32-e03505-s002.pdf]

Supporting Information

A Mononuclear Scenario for the Copper–Catalyzed Monooxygenation of Phenolic Substrates

Alexander Koch,[a] Antony Memboeuf,[b] Felix Tuczek*[a] and Tobias A. Engesser*[a]

[a] Dr. A. Koch, Prof. Dr. F. Tuczek, Dr. T. A. Engesser
Institut für Anorganische Chemie
Christian-Albrechts-Universität zu Kiel
Max-Eyth-Straße 2, 24118 Kiel, Germany
E-mail: ftuczek@ac.uni-kiel.de
tengesser@ac.uni-kiel.de

[b] Dr. A. Memboeuf
Univ. Brest, CNRS, UMR 6521, F 29200 Brest, France
E-mail: antony.memboeuf@univ-brest.fr

Content

1. Computational Details 2
2. Validation of method 2
3. Thermodynamics 4
   3.1. Analysis of possible isomers 5
   3.2. Entropies, Enthalpies and Free Energies 7
4. NMR spectrometry 23
5. Mass spectrometry 24
6. References 26

1. Computational Details

Calculations of the structures, entropies, enthalpies and free energies were performed using the ORCA 4.2.1 program package[1] on PBE0[2]/def2-TZVPP[3] level with Grimmes dispersion correction,[4] Becke-Johnson damping (D3BJ),[5] density fitting approximation (RIJCOSX),[6] and with solvation correction (CPCM).[7] All calculated structures were proofed to be energetic minima by frequency analysis, showing no imaginary frequencies. As solvent a DCM employed with a dielectric constant of 8.93, as it is usually employed for the catalytic experiments with bidentate triazoles,[8,9] BPM[10] or DPM.[11]

Transition states (TS) are found via the minimum energy path and the saddle point connecting two minimum structures with the *Nudged Elastic Band method*, which is implemented as tool in the ORCA program package (NEB-TS).[12] At first coordinates of the desired transition state were obtained on BP86[13]/def2-SVP[14] level and then used as guess for a PBE0-D3(BJ)/def2-TZVPP calculation. The gas phase structure of the transition state was then used for a single point calculation in solution to obtain the Gibbs free energy of the TS in DCM solution.

2. Validation of method

Previous studies showed that hybrid DFT functionals (e.g. B3LYP) are sufficiently accurate for the copper systems presented herein.[15] Furthermore, there seem to be small differences between the accuracy of the different functionals, for instance for first row transition metal systems[16] and compared to the more modern QM/MM hybrid methods and especially for large systems, DFT is still able to provide accurate results, and at a relatively low computational cost.[17] Therefore, and due to the large quantity of intermediates and transition states described in this study, non-empirical global hybrid generalized gradient approximation functional PBE0 was chosen for the thermodynamic calculations of the copper complexes, which offers a good balance between accuracy and computational cost, and has been shown to perform reliably for transition metal systems, including copper. Compared to other hybrid functionals PBE0 generally provides improved accuracy in electronic structure and energetic predictions without the need for extensive empirical parameterization. Furthermore, the inclusion of 25% exact Hartree–Fock exchange in PBE0

helps to mitigate self-interaction errors, which are particularly relevant in systems with open-shell transition metals, such as the complexes in this study. The combination with def2-TZVPP as basis set also reproduced the structural differences in copper systems related to this study well. (**Figure S1**) As the catalytic experiments with the chosen systems usually proceed in DCM, all calculations were performed with solvation correction (CPCM, DCM, ε = 8.93) and as they contain relatively large substrate and ligand molecules and also aromatic systems also D3(BJ) dispersion correction was included.

To validate the used method (PBE0-D3(BJ)/def2-TZVPP) and ensure reliability in the analysis, the structures of two known complexes similar to the ones investigated in this study were calculated. As the experimental bond lengths are reproduced with good accuracy, which was checked by comparison to available X-ray diffraction (XRD) data (**Figure S1**), this method was deemed to be sufficiently accurate.

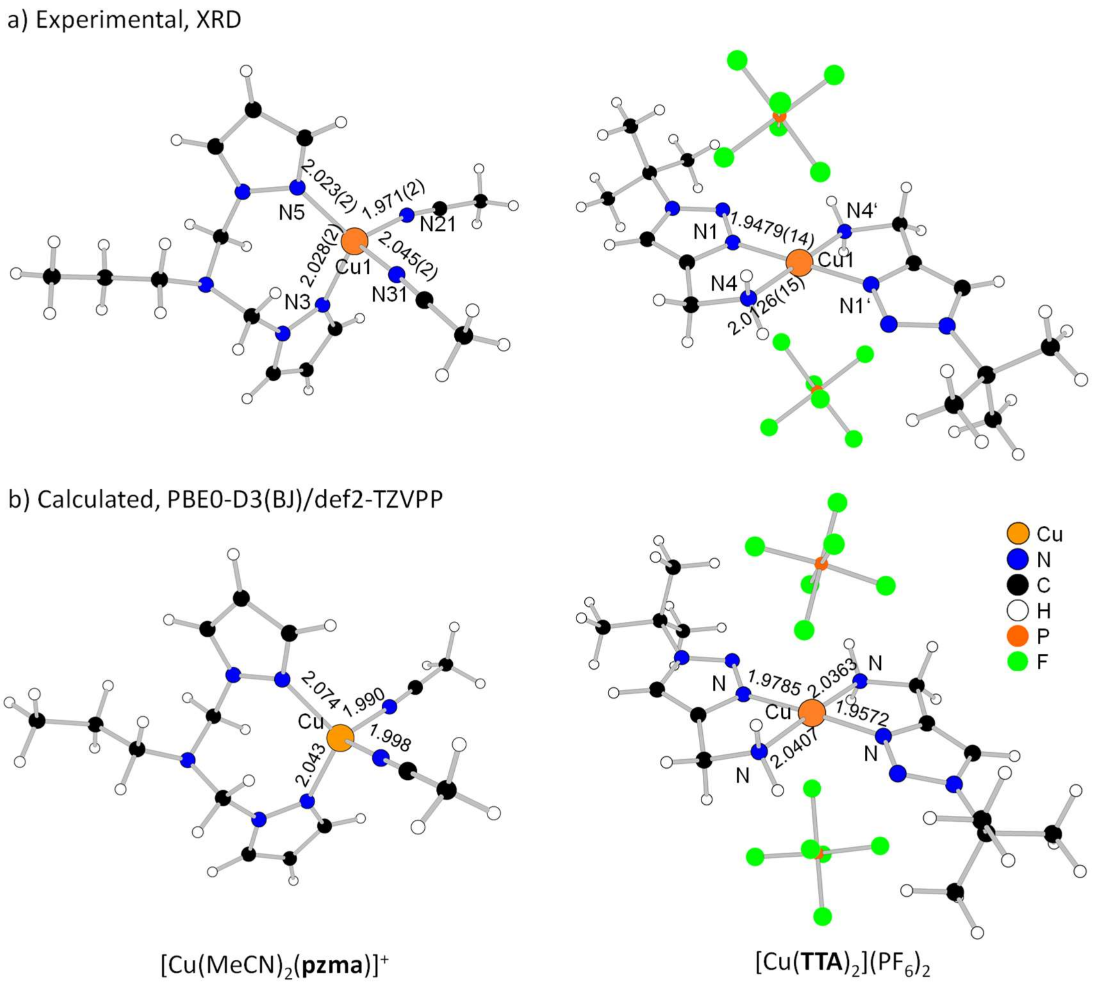

Figure S1. a) Experimental XRD and b) theoretical structures optimized with PBE0-D3(BJ)/def2-TZVPP of $[Cu(MeCN)_2($**pzma**$)]^+$ [18] and $[Cu($**TTA**$)_2](PF_6)_2$ [8] for validation of the method.

3. Thermodynamics

As the cycle starts with a copper(**I**) complex and the supporting ligands, the substrate **DMP-H** and added O_2 are neutral, all the relevant intermediates and transition states are singly positively charged and have a spin of S = $^1/_2$ (doublet). Additionally, starting with a S = $^3/_2$ (quartet) dioxygen complex (**O$_2$-P**) was excluded by comparison of the corresponding free energies.

3.1. Analysis of possible isomers

The effect of the different possible isomers is discussed below using **MTA** complexes as example.

The coordination of the asymmetric bidentate ligands can lead to two different coordination isomers. Starting the reaction mechanism with a dioxygen phenolate complex (**O_2-P**), it makes sense to distinguish the two isomers by their coordination in relation to the O_2 coligand. Therefore, we decided to refer to them as complexes with linkage isomers including the triazole (**MTA**-*trans*-triazole, **Figure S2**, a and b, and **Table S1**) or the amine *trans* (**MTA**-*trans*-amine, **Figure S2**, c and d, and **Table S1**) to the dioxygen ligand. Furthermore, here the rather flexible methylamine group $-CH_2NH_2$ can lead to two different conformational isomers with the $-CH_2NH_2$ group facing up- or downwards in relation to the plane of the copper coordination sphere (**Figure S2**).

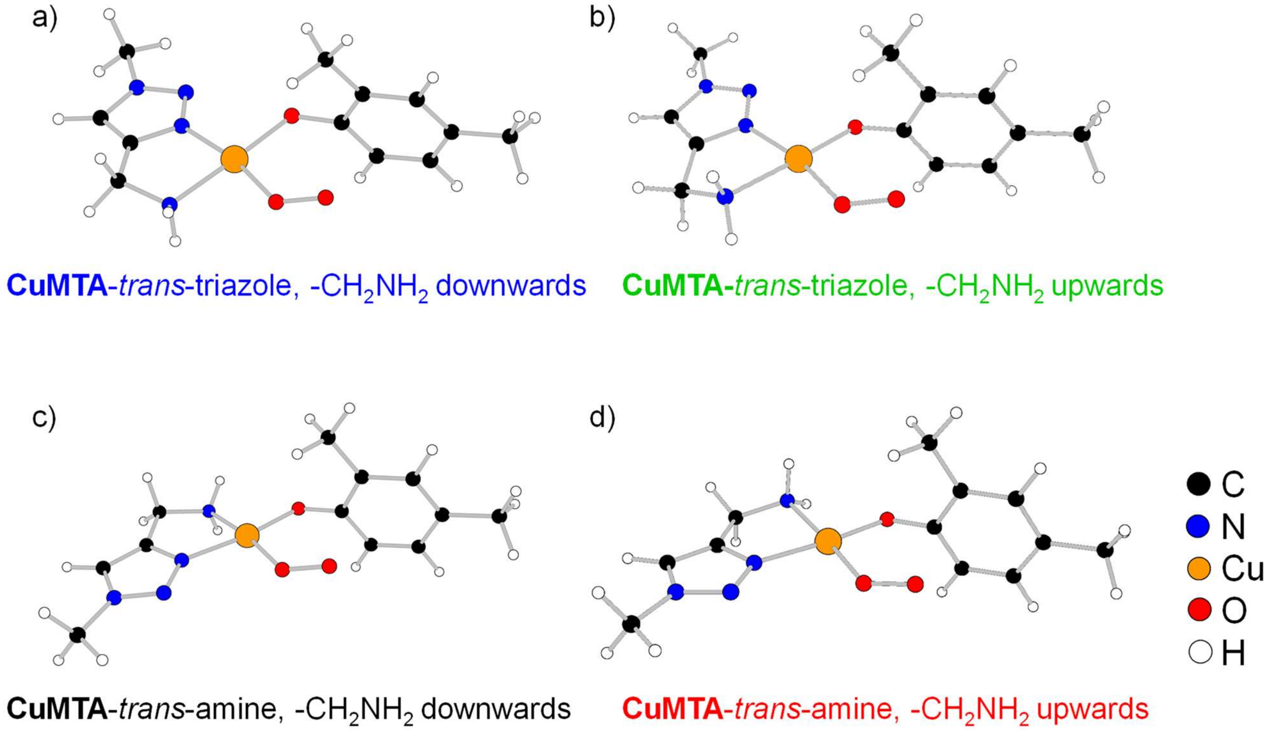

Figure S2. Linkage isomers of **CuMTA** dioxygen phenolate complexes (**O_2-P**) including conformational isomers with the $-CH_2NH_2$ group facing down- (a) and c)) or upwards (b) and d)) in relation to the plane of the copper coordination sphere.

When examining the structures of the dioxygen phenolate complexes with **MTA** as a ligand (**Figure S2**), it appears that the conformation has little impact on the overall structure and,

consequently, on the energetics of these molecules. This is indeed true for the square planar geometry observed in this case. The same holds for the metallacyclic complexes (**M**, **Figure S3**). However, a different pattern emerges when looking at the corresponding hydroxo complexes, which adopt a square pyramidal geometry (**H-Q**, **Figure S3**). When comparing the two conformational isomers of the hydroxo complexes in which the triazole group coordinates *trans* to the OH group with those with a *trans*-standing amine, one can see that the latter have a slightly higher energy by around 5-10 $kJ\cdot mol^{-1}$. This has most likely to do with a more favorable (less bend) coordination mode of the quinone.

In summary, for **MTA** and **MTEA** it seems to be more favorable to form **M** and **H-Q** when **MTA** is coordinating with a trans-standing triazole, therefore, these versions are included in the energetics presented in the main article. If the $-CH_2NH_2$ group is facing up- or downwards seems to have only a marginal effect on the energetics, therefore, without any specific reason we decided to use the data with the $-CH_2NH_2$ group upwards for **CuMTA** and for **CuMTEA** with a *trans*-standing amine for the discussion, for **CuMTEA** with a *trans*-standing triazole the $-CH_2NH_2$ group stands down, but all data was obtained for all versions. For the tables only two versions are listed for all four ligands.

For **CuBPM M** and **H-Q** were more stable with conformer 1 and for **CuDPM H-Q** is much more stable with conformer 1, therefore in both cases conformer 1 was included in the main article (**Figure 1**).

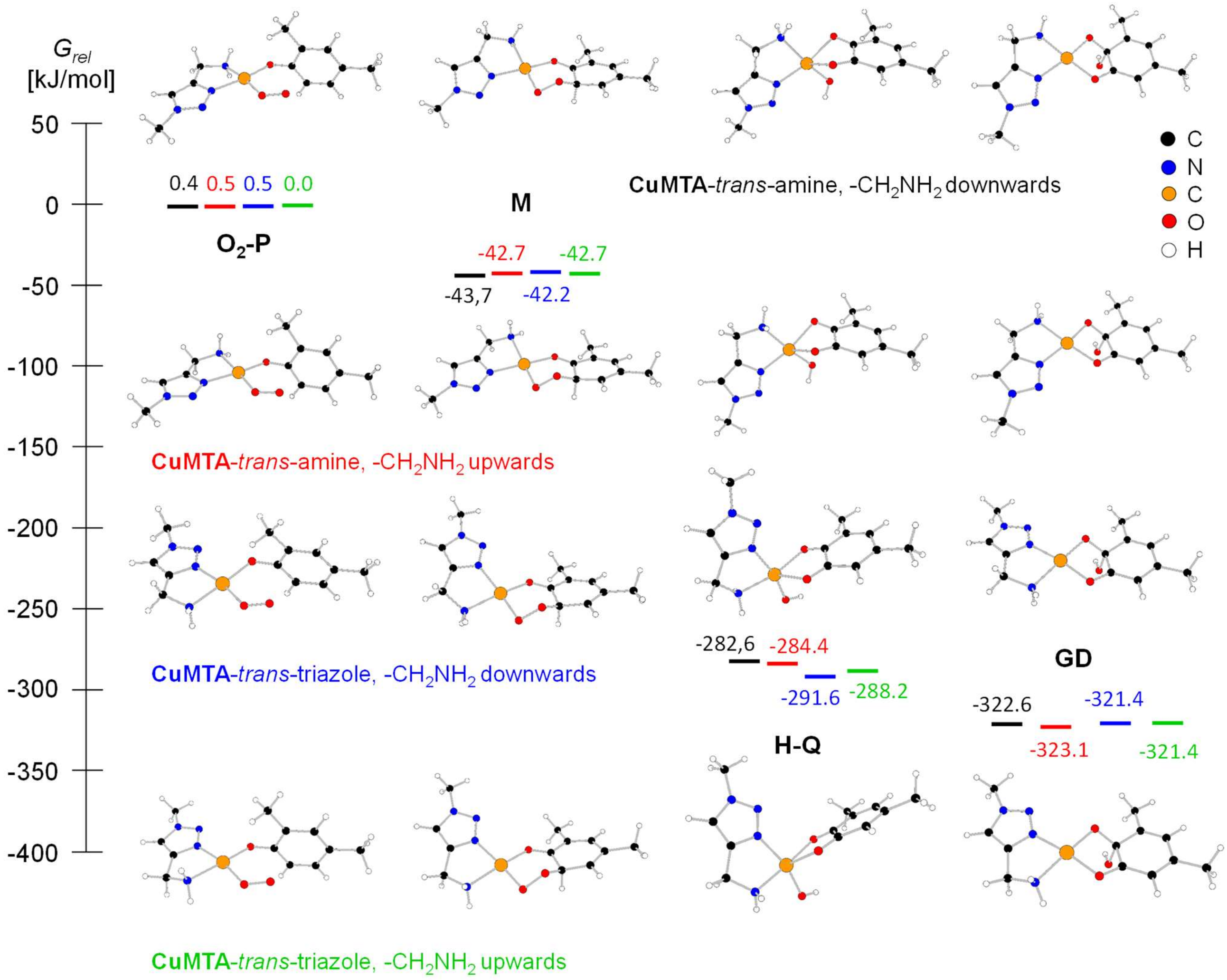

Figure S3. Conformational analysis for **CuMTA** including *trans*-standing amine with -CH_2NH_2 group downwards (black) and upwards (red) as well as *trans*-standing triazole with -CH_2NH_2 group downwards (blue) and upwards (green) (*trans* to dioxygen ligand in **O_2-P**) by comparison of the relative free energies G_{rel} [kJ·mol^{-1}] of the molecules.

3.2. Entropies, Enthalpies and Free Energies

Entropies (S), enthalpies (H) and free energies (G) of the dioxygen phenolate (**O_2-P**), metallacyclic (**M**), hydroxo quinone (**H-Q**) and geminal diolate complexes (**GD**) as well as the transitions state between them (**$TS^1_{O2\text{-}P/M}$**, **$TS^2_{M/H\text{-}Q}$** and **$TS^3_{M/HA}$**) were all calculated on BP86/def2-SVP and PBE0-D3(BJ)/def2-TVPP level as well as with PBE0-D3(BJ)/def2-TVPP in solution with all four presented ligands (**TTA, MTA**, **MTEA**, **BPM** and **DPM**). The results are listed in the following Tables (**Table S1**, **Table S2**, **Table S3, Table S4** and **Table**

S5). In case of **CuMTA** and **CuMTEA** only the more stable conformational isomers are listed, which are the ones with the -CH_2NH_2 group standing up for **CuMTA** (cf. Figure S2) and the *trans*-amine complexes with -CH_2NH_2 group standing up and *trans*-triazole complexes with -$CH_2CH_2NH_2$ group standing down for **CuMTEA**. For **CuBPM** and **CuDPM**, the two conformers with the -NH_2- bridge facing up- or downwards in relation to the equatorial CuN_2O_2 plane. After the conformational analysis for **CuMTA** and **CuMTEA**, in case of **CuTTA**, only the energies of the most stable conformer, which is the *trans*-triazole with the -CH_2NH_2 group standing up was included and listed.

Table S1: Calculated entropies, enthalpies and free energies at 298.15K of **CuMTA** complexes on BP86/def2-SVP and PBE0/def2-TZVPP level including dispersion (D3(BJ)) and solvation correction (CPCM, DCM, ε = 8.93) in [kJ·mol^{-1}].

**Ligand**-coordination mode	BP86/def2-SVP-D3(BJ)			PBE0/def2-TZVPP-D3(BJ)			PBE0/def2-TZVPP-D3(BJ) in solution			
**MTA**-trans-triazole[b)]	$T{*}S_{(g)}$	$H_{(g)}$	$G_{(g)}$	$T{*}S_{(g)}$	$H_{(g)}$	$G_{(g)}$	$T{*}S_{(solv)}$	$H_{(solv)}$	$G_{(solv)}$	$\boldsymbol{G_{rel(solv)}}$[a)]
**O_2-P**	-187,793	-6699188,933	-6699376,726	-190,656	-6698362,958	-6698362,958	-173,313	-6698382,853	-6698556,166	**0**
**M**	-188,457	-6699178,417	-6699366,874	-184,293	-6698226,202	-6698410,495	-165,046	-6698433,819	-6698598,865	**-42,70**
**H-Q**	-190,158	-6699389,788	-6699579,946	-191,907	-6698451,767	-6698643,675	-179,454	-6698664,881	-6698844,336	**-288,17**
**GD**	-186,873	-6699406,872	-6699593,745	-183,384	-6698489,037	-6698672,421	-172,337	-6698705,249	-6698877,586	**-321,42**
T**S$^{1}_{O2\text{-}P/M}$**	-189,018	-6699150,924	-6699339,942	-186,020	-6697138,880	-6698324,900	-171,576	-6698340,498	-6698512,074	**44,09**
T**S$^{2}_{M/H\text{-}Q}$**	-186,085	-6699108,567	-6699294,652	-181,287	-6698142,966	-6698324,253	-171,594	-6698352,214	-6698523,809	**32,36**
T**S$^{3}_{H\text{-}Q/GD}$**	-188,671	-6699383,296	-6699571,967	-181,739	-6698437,629	-6698619,367	-182,137	-6698638,396	-6698820,533	**-264,37**
**MTA**-trans-amine[c)]	$T{*}S_{(g)}$	$H_{(g)}$	$G_{(g)}$	$T{*}S_{(g)}$	$H_{(g)}$	$G_{(g)}$	$T{*}S_{(solv)}$	$H_{(solv)}$	$G_{(solv)}$	$\boldsymbol{G_{rel(solv)}}$[a)]
**O_2-P**	-193,025	-6699195,256	-6699388,281	-191,852	-6698181,656	-6698373,508	-166,925	-6698555,635	-6698555,635	**0**
**M**	-187,802	-6699171,825	-6699359,626	-184,075	-6698217,157	-6698401,232	-170,507	-6698428,314	-6698598,822	**-43,19**
**H-Q**	-190,763	-6699288,867	-6699579,630	-190,196	-6698446,111	-6698636,307	-180,322	-6698660,254	-6698840,577	**-284,94**
**GD**	-186,598	-6699409,327	-6699595,925	-183,381	-6698493,921	-6698677,303	-171,902	-6698707,390	-6698879,292	**-323,66**
T**S$^{1}_{O2\text{-}P/M}$**	-185,235	-6699170,684	-6699355,920	-186,144	-6698140,193	-6698326,337	-178,413	-6698336,388	-6698514,801	**40,83**
T**S$^{2}_{M/H\text{-}Q}$**	-185,477	-6699112,495	-6699297,973	-181,250	-6698145,823	-6698327,074	-169,060	-6698350,440	-6698519,499	**36,14**
T**S$^{3}_{H\text{-}Q/GD}$**	-185,045	-6699384,969	-6699570,014	-182,469	-6698445,002	-6698627,472	-182,042	-6698643,526	-6698825,569	**-269,93**

a) The energy relative to the dioxygen phenolate complex (**O_2-P**) is given. b) Triazole group of the bidentate ligand coordinating *trans* to O_2 and -CH_2NH_2 group standing up (Figure 2). c) Amine group of the bidentate ligand coordinating trans to O_2 and -CH_2NH_2 group standing up (Figure S4).

Table S2: Calculated entropies, enthalpies and free energies at 298.15K of **CuMTEA** complexes on BP86/def2-SVP and PBE0/def2-TZVPP level including dispersion (D3(BJ)) and solvation correction (CPCM, DCM, ε = 8.93) in [kJ·mol^{-1}].

**Ligand**-coordination mode	BP86/def2-SVP-D3(BJ)			PBE0/def2-TZVPP-D3(BJ)			PBE0/def2-TZVPP-D3(BJ) in solution (CPCM, DCM, ε = 8.93)			
**MTEA**-trans-triazole[b)]	$T*S_{(g)}$	$H_{(g)}$	$G_{(g)}$	$T*S_{(g)}$	$H_{(g)}$	$G_{(g)}$	$T*S_{(solv)}$	$H_{(solv)}$	$G_{(solv)}$	$\boldsymbol{G_{rel(solv)}}$[a)]
**O_2-P**	-200,574	-6802288,542	-6802489,116	-196,364	-6801253,181	-6801449,545	-178,636	-6801457,360	-6801635,996	**0**
**M**	-193,404	-6802284,974	-6802478,378	-189,364	-6801307,950	-6801497,314	-176,443	-6801505,075	-6801681,518	**-45,52**
**H-Q**	-197,071	-6802493,233	-6802690,304	-197,434	-6801533,565	-6801730,999	-184,609	-6801740,085	-6801924,695	**-288,70**
**GD**	-193,116	-6802513,401	-6802706,517	-189,263	-6801571,818	-6801761,080	-177,001	-6801779,543	-6801956,543	**-320,55**
T**S^1$_{O2-P/M}$**	-193,741	-6802253,893	-6802447,634	-192,072	-6801219,496	-6801411,568	-177,460	-6801413,271	-6801590,731	**45,26**
T**S^2$_{M/H-Q}$**	-191,152	-6802216,086	-6802407,238	-186,925	-6801224,758	-6801411,683	-174,593	-6801424,735	-6801599,328	**36,67**
T**S^3$_{H-Q/GD}$**	-191,836	-6802484,545	-6802676,381	-187,197	-6801523,935	-6801711,132	-186,724	-6801716,78	-6801903,504	**-267,51**
**MTEA**-trans-amine[c)]	$T*S_{(g)}$	$H_{(g)}$	$G_{(g)}$	$T*S_{(g)}$	$H_{(g)}$	$G_{(g)}$	$T*S_{(solv)}$	$H_{(solv)}$	$G_{(solv)}$	$\boldsymbol{G_{rel(solv)}}$[a)]
**O_2-P**	-198,554	-6802300,682	-6802499,235	-194,191	-6801266,112	-6801460,303	-176,900	-6801461,021	-6801637,921	**0**
**M**	-194,062	-6802275,988	-6802470,051	-189,750	-6801298,413	-6801488,164	-176,734	-6801502,742	-6801679,476	**-41,55**
**H-Q**	-197,673	-6802498,217	-6802695,890	-194,757	-6801530,674	-6801723,431	-184,068	-6801734,250	-6801918,319	**-280,40**
**GD**	-191,587	-6802514,469	-6802706,056	-188,587	-6801572,312	-6801760,899	-175,680	-6801781,520	-6801957,200	**-319,28**
T**S^1$_{O2-P/M}$**	-195,939	-6802257,785	-6802453,724	-193,104	-6801220,792	-6801413,896	-178,543	-6801411,610	-6801590,153	**47,77**
T**S^2$_{M/H-Q}$**	-191,241	-6802220,149	-6802411,389	-187,620	-6801230,103	-6801417,723	-179,574	-6801423,602	-6801603,176	**34,74**
T**S^3$_{H-Q/GD}$**	-191,479	-6802496,995	-6802688,474	-188,401	-6801530,861	-6801719,262	-175,291	-6801728,874	-6801904,165	**-266,24**

a) The energy relative to the dioxygen phenolate complex (**O_2-P**) is given. b) Triazole group of the bidentate ligand coordinating trans to O_2 (Figure S5). c) Amine group of the bidentate ligand coordinating trans to O_2 (Figure S6).

Table S3: Calculated entropies, enthalpies and free energies at 298.15K of **CuBPM** complexes on BP86/def2-SVP and PBE0/def2-TZVPP level including dispersion (D3(BJ)) and solvation correction (CPCM, DCM, ε = 8.93) in [kJ·mol^{-1}].

**Ligand-** coordination mode	BP86/def2-SVP-D3(BJ)			PBE0/def2-TZVPP-D3(BJ)			PBE0/def2-TZVPP-D3(BJ) in solution (CPCM, DCM, ε = 8.93)			
**BPM**-conformer1[b)]	$T^*S_{(g)}$	$H_{(g)}$	$G_{(g)}$	$T^*S_{(g)}$	$H_{(g)}$	$G_{(g)}$	$T^*S_{(solv)}$	$H_{(solv)}$	$G_{(solv)}$	$\boldsymbol{G_{rel(solv)}}$[a)]
**O₂-P**	-199,706	-6999095,257	-6999294,963	-195,788	-6998014,182	-6998209,969	-179,223	-6998198,960	-6998378,183	**0**
**M**	-192,803	-6999080,345	-6999273,148	-187,996	-6998059,167	-6998247,163	-177,828	-6998246,501	-6998424,329	**-46,15**
**H-Q**	-195,159	-6999285,567	-6999480,726	-195,678	-6998277,306	-6998472,984	-192,045	-6998476,052	-6998668,098	**-289,91**
**GD**	-190,939	-6999316,268	-6999507,207	-187,439	-6998331,967	-6998519,406	-184,892	-6998520,623	-6998705,515	**-327,33**
T**S^1$_{\mathbf{O2\text{-}P/M}}$**	-193,127	-6999056,581	-6999249,708	-189,852	-6997978,310	-6998168,162	-179,831	-6998158,291	-6998338,122	**40,06**
T**S^2$_{\mathbf{M/H\text{-}Q}}$**	-190,372	-6999012,264	-6999202,636	-184,987	-6997982,279	-6998167,266	-176,146	-6998165,820	-6998341,967	**36,22**
T**S^3$_{\mathbf{H\text{-}Q/GD}}$**	-189,680	-6999279,871	-6999469,550	-186,896	-6998275,362	-6998462,258	-184,038	-6998461,193	-6998645,231	**-267,05**
**BPM**-conformer2[c)]	$T^*S_{(g)}$	$H_{(g)}$	$G_{(g)}$	$T^*S_{(g)}$	$H_{(g)}$	$G_{(g)}$	$T^*S_{(solv)}$	$H_{(solv)}$	$G_{(solv)}$	$T^*S_{(g)}$
**O₂-P**	-198,149	-6999096,728	-6999294,877	-195,005	-6998013,617	-6998208,621	-184,839	-6998197,424	-6998382,262	**0**
**M**	-192,698	-6999083,108	-6999275,806	-188,225	-6998060,913	-6998249,139	-177,560	-6998247,845	-6998425,405	**-43,14**
**H-Q**	-195,256	-6999292,861	-6999488,117	-194,754	-6998276,644	-6998471,398	-193,222	-6998471,913	-6998665,136	**-282,87**
**GD**	-191,192	-6999316,696	-6999507,888	-187,474	-6998332,779	-6998520,253	-179,734	-6998523,296	-6998703,030	**-320,76**
T**S^1$_{\mathbf{O2\text{-}P/M}}$**	-193,029	-6999059,230	-6999282,259	-190,171	-6997977,357	-6998167,528	-179,858	-6998158,972	-6998338,830	**43,43**
T**S^2$_{\mathbf{M/H\text{-}Q}}$**	-190,229	-6999015,420	-6999205,649	-185,644	-6997984,956	-6998170,600	-175,663	-6998166,029	-6998341,692	**40,57**
T**S^3$_{\mathbf{H\text{-}Q/GD}}$**	-189,281	-6999286,538	-6999475,819	-186,621	-6998276,102	-6998462,722	-184,180	-6998462,208	-6998646,388	**-264,12**

a) The energy relative to the dioxygen phenolate complex (**O₂-P**) is given. b) -NH_2- bridge standing up in relation to the equatorial CuN_2O_2 plane (Figure S7). c) -NH_2- bridge standing down in relation to the equatorial CuN_2O_2 plane (Figure S8).

Table S4: Calculated entropies, enthalpies and free energies at 298.15K of **CuDPM** complexes on BP86/def2-SVP and PBE0/def2-TZVPP level including dispersion (D3(BJ)) and solvation correction (CPCM, DCM, ε = 8.93) in [kJ·mol^{-1}].

**Ligand**-coordination mode	BP86/def2-SVP-D3(BJ)			PBE0/def2-TZVPP-D3(BJ)			PBE0/def2-TZVPP-D3(BJ) in solution (CPCM, DCM, ε = 8.93)			
**DPM**-conformer1[b)]	$T*S_{(g)}$	$H_{(g)}$	$G_{(g)}$	$T*S_{(g)}$	$H_{(g)}$	$G_{(g)}$	$T*S_{(solv)}$	$H_{(solv)}$	$G_{(solv)}$	$\boldsymbol{G_{rel(solv)}}$[a)]
**O_2-P**	-199,924	-7114935,567	-7115135,492	-202,208	-7113799,101	-7114001,309	-191,939	-7113969,016	-7114000,630	**0**
**M**	-200,382	-7114915,981	-7115116,363	-195,792	-7113840,042	-7114035,834	-190,797	-7114011,507	-7114202,304	**-41,35**
**H-Q**	-202,152	-7115128,455	-7115330,607	-201,986	-7114064,995	-7114266,981	-197,749	-7114245,939	-7114443,688	**-282,73**
**GD**	-192,426	-7115161,374	-7115353,800	-194,905	-7114118,141	-7114343,046	-191,380	-7114290,986	-7114482,366	**-321,41**
T**S$^1_{O2-P/M}$**	-199,388	-7114894,253	-7115093,641	-195,944	-7113761,832	-7113957,776	-185,654	-7113927,627	-7114113,281	**47,67**
T**S$^2_{M/H-Q}$**	-197,530	-7114853,940	-7115051,470	-189,094	-7113759,553	-7113948,647	-184,983	-7113924,575	-7114109,558	**51,40**
T**S$^3_{H-Q/GD}$**	-197,162	-7115130,161	-7115327,323	-188,058	-7114043,694	-7114231,752	-190,728	-7114209,856	-7114400,584	**-239,63**
**DPM**-conformer2[c)]	$T*S_{(g)}$	$H_{(g)}$	$G_{(g)}$	$T*S_{(g)}$	$H_{(g)}$	$G_{(g)}$	$T*S_{(solv)}$	$H_{(solv)}$	$G_{(solv)}$	$\boldsymbol{G_{rel(solv)}}$[a)]
**O_2-P**	-204,738	-7114934,325	-7115139,064	-202,156	-7113798,473	-7114000,630	-192,408	-7113967,691	-7114160,099	**0**
**M**	-199,472	-7114920,805	-7115120,277	-195,072	-7113844,573	-7114039,645	-190,838	-7114013,903	-7114204,741	**-44,64**
**H-Q**	-201,732	-7115138,442	-7115340,173	-199,997	-7114066,375	-7114266,372	-191,858	-7114236,331	-7114428,189	**-268,09**
**GD**	-198,671	-7115160,276	-7115358,948	-194,556	-7114118,534	-7114313,090	-191,946	-7114290,120	-7114482,066	**-321,97**
T**S$^1_{O2-P/M}$**	-199,223	-7114895,372	-7115094,595	-198,524	-7113759,014	-7113957,537	-187,164	-7113925,397	-7114112,561	**47,54**
T**S$^2_{M/H-Q}$**	-197,117	-7114858,073	-7115055,190	-187,002	-7113770,513	-7113957,515	-189,441	-7113930,074	-7114119,515	**40,58**
T**S$^3_{H-Q/GD}$**	-196,134	-7115136,591	-7115332,724	-194,649	-7114066,941	-7114261,590	-190,315	-7114234,598	-7114424,913	**-264,81**

a) The energy relative to the dioxygen phenolate complex (**O_2-P**) is given. b) -NH_2- bridge standing up in relation to the equatorial CuN_2O_2 plane (Figure S9). c) -NH_2- bridge standing down in relation to the equatorial CuN_2O_2 plane (Figure S10).

Table S5: Calculated entropies, enthalpies and free energies at 298.15K of **CuTTA** complexes on BP86/def2-SVP and PBE0/def2-TZVPP level including dispersion (D3(BJ)) and solvation correction (CPCM, DCM, ε = 8.93) in [kJ·mol^{-1}].

**Ligand**-coordination mode	BP86/def2-SVP-D3(BJ)			PBE0/def2-TZVPP-D3(BJ)			PBE0/def2-TZVPP-D3(BJ) in solution			
**TTA**-trans-triazole[b)]	$T*S_{(g)}$	$H_{(g)}$	$G_{(g)}$	$T*S_{(g)}$	$H_{(g)}$	$G_{(g)}$	$T*S_{(solv)}$	$H_{(solv)}$	$G_{(solv)}$	$\mathbf{G_{rel(solv)}}$[a)]
**O_2-P**	-216,479	-7008444,355	-7008660,834	-210,596	-7007373,803	-7007584,400	-191,071	-7007575,303	-7007766,375	**0**
**M**	-208,577	-7008434,927	-7008643,505	-204,196	-7007425,657	-7007629,853	-183,354	-7007624,337	-7007807,691	**-41,32**
**H-Q**	-210,279	-7008642,302	-7008852,581	-212,074	-7007650,962	-7007863,036	-197,059	-7007861,082	-7008058,140	**-291,77**
**GD**	-205,510	-7008657,696	-7008863,206	-203,223	-7007690,840	-7007894,063	-193,011	-7007898,069	-7008091,081	**-324,71**
T**S^1$_{O2-P/M}$**	-201,001	-7008410,351	-7008611,352	-205,183	-7007339,962	-7007545,145	-195,676	-7007530,658	-7007726,333	**40,04**
T**S^2$_{M/H-Q}$**	-201,001	-7008367,476	-7008572,686	-201,772	-7007343,904	-7007545,676	-188,905	-7007541,662	-7007730,567	**35,81**
T**S^3$_{H-Q/GD}$**	-204,176	-7008637,916	-7008842,093	-202,225	-7007639,486	-7007841,711	-194,875	-7007833,135	-7008028,010	**-261,64**

a) The energy relative to the dioxygen phenolate complex (**O_2-P**) is given. b) Triazole group of the bidentate ligand coordinating *trans* to O_2 and -CH_2NH_2 group standing up. Amine group of the bidentate ligand coordinating trans to O_2 and -CH_2NH_2 group standing up (see Figure S11).

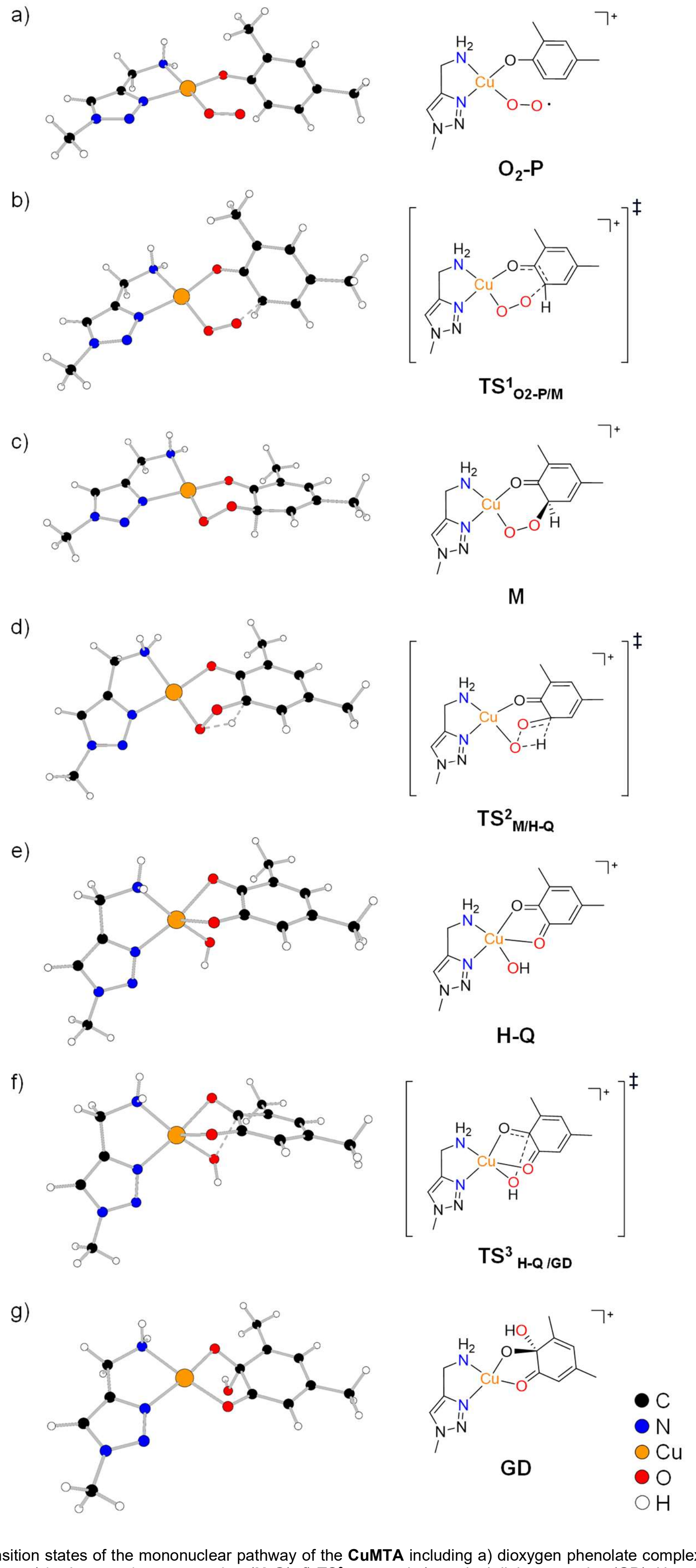

Figure S4. Intermediates and transition states of the mononuclear pathway of the **CuMTA** including a) dioxygen phenolate complex (**O_2-P**) b) **$TS^1_{O2\text{-}P/M}$**, c) metallacyclic complex (**M**) d) **$TS^2_{M/H\text{-}Q}$** e) hydroxo quinone complex (**H-Q**), f) **$TS^3_{H\text{-}Q/GD}$** and g) geminal diolate complex (**GD**). Here, the amine donor of the **MTA** ligand coordinates *trans* to dioxygen ligand in **O_2-P**, in contrast to the structures with a *trans*-standing triazole in Figure 2.

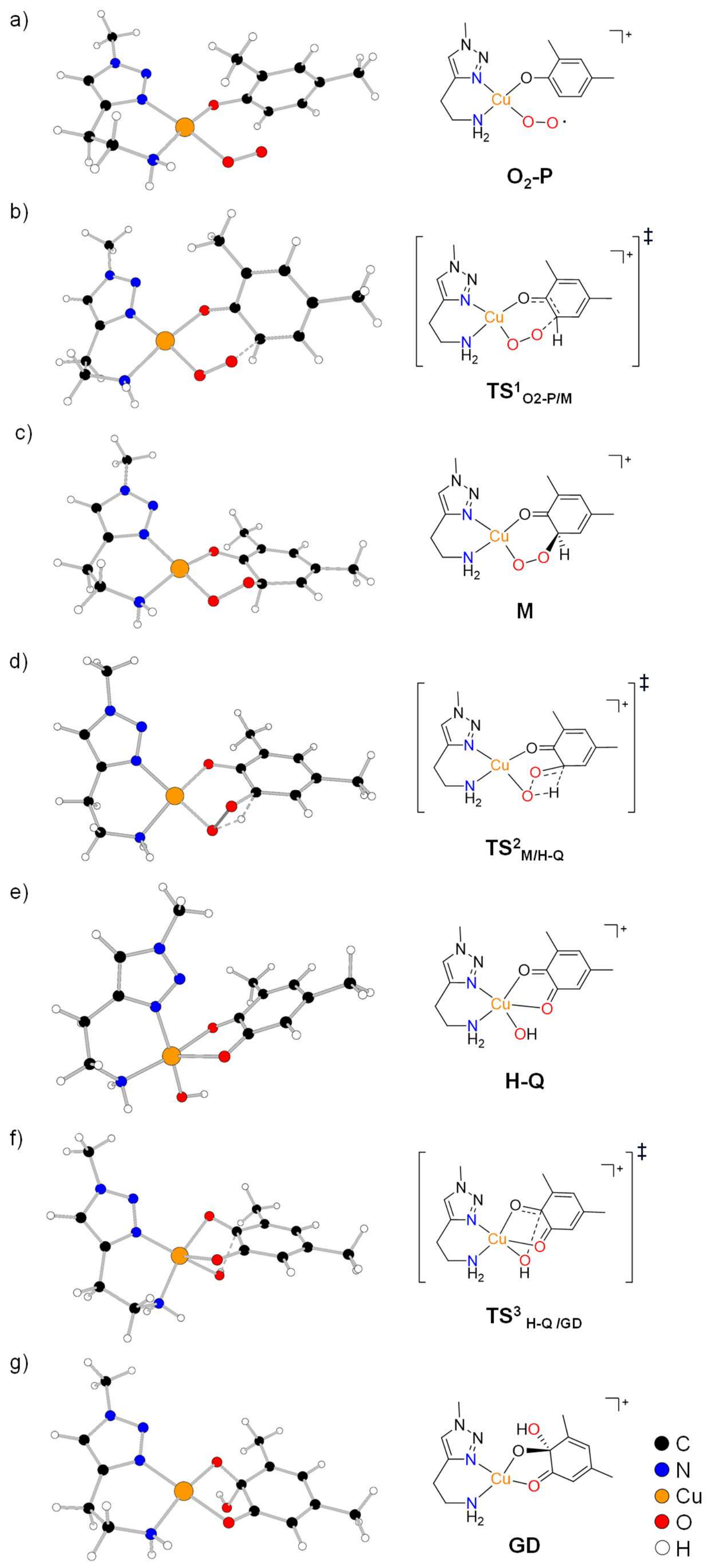

Figure S5. Intermediates and transition states of the mononuclear pathway of the **CuMTEA** system including dioxygen phenolate complex (**O_2-P**) b) **$TS^1_{O2\text{-}P/M}$**, c) metallacyclic complex (**M**) d) **$TS^2_{M/H\text{-}Q}$** e) hydroxo quinone complex (**H-Q**), f) **$TS^3_{H\text{-}Q/GD}$** and g) geminal diolate complex (**GD**). Here, the triazole donor of the **MTEA** ligand coordinates *trans* to dioxygen ligand in **O_2-P**, in contrast to the structures with a *trans*-standing triazole in Figure S6.

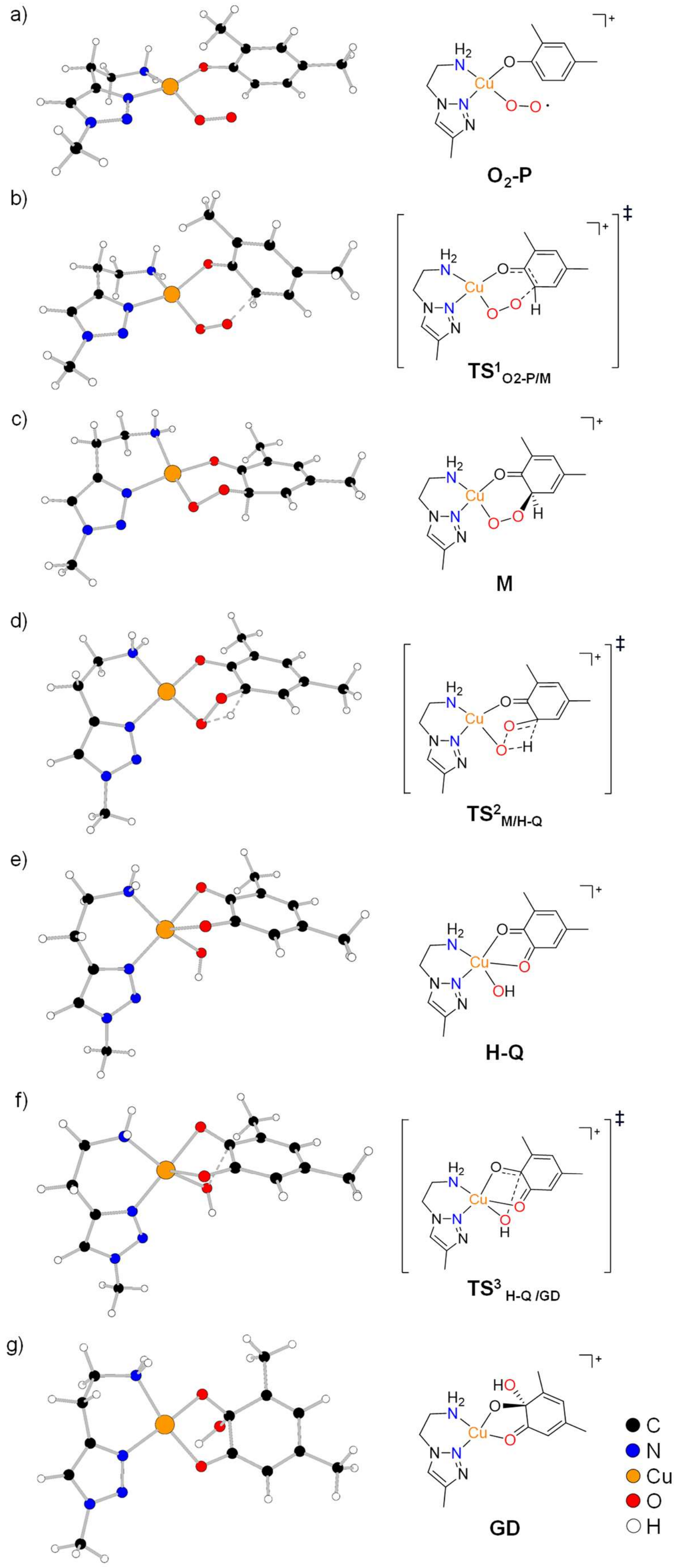

Figure S6. Intermediates and transition states of the mononuclear pathway of the **CuMTEA** system including a) dioxygen phenolate complex (**O_2-P**) b) **$TS^1_{O2\text{-}P/M}$**, c) metallacyclic complex (**M**) d) **$TS^2_{M/H\text{-}Q}$** e) hydroxo quinone complex (**H-Q**), f) **$TS^3_{H\text{-}Q/GD}$** and g) geminal diolate complex (**GD**). Here, the amine donor of the **MTEA** ligand coordinates *trans* to dioxygen ligand in **O_2-P**, in contrast to the structures with a *trans*-standing triazole in Figure S5.

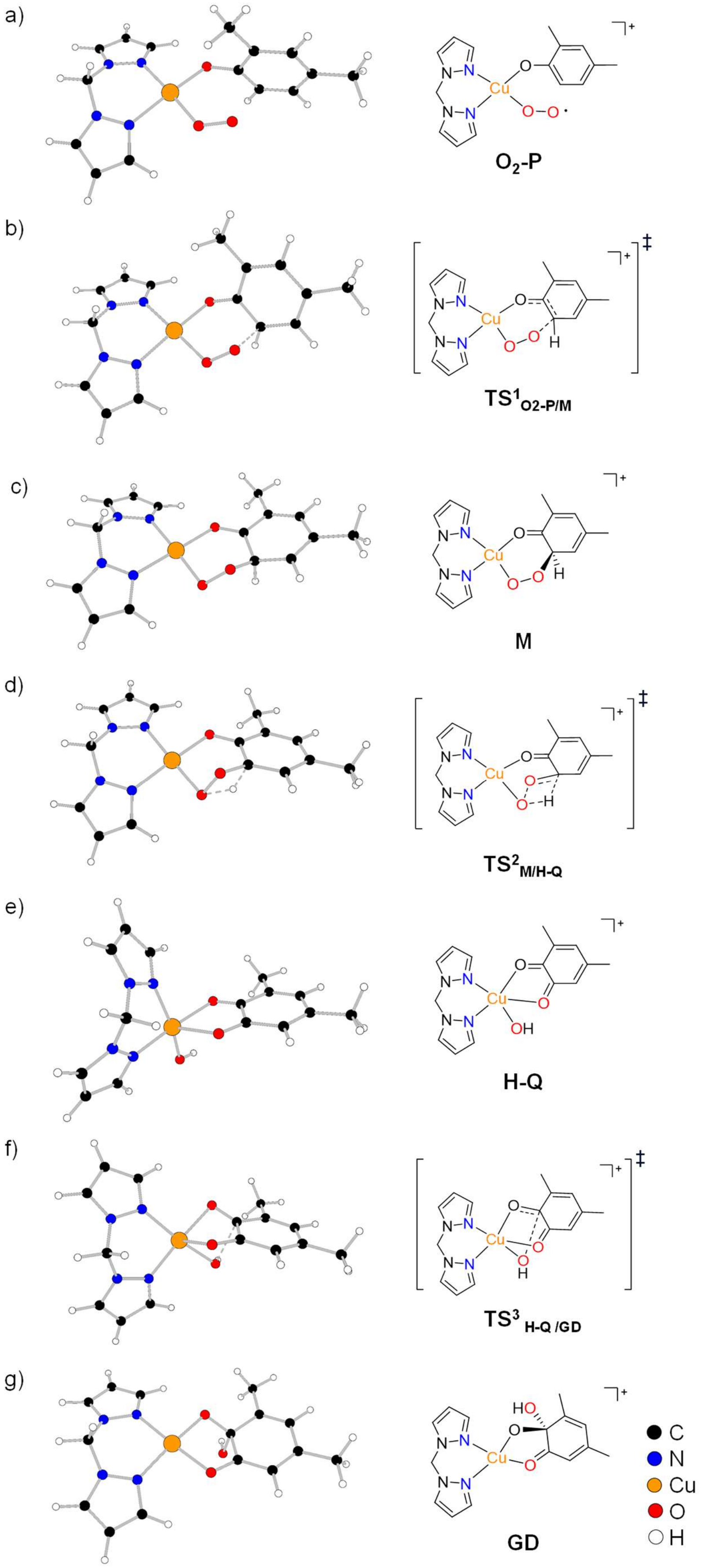

Figure S7. Intermediates and transition states of the mononuclear pathway of the **CuBPM** system including a) dioxygen phenolate complex (**O_2-P**) b) **$TS^1_{O2\text{-}P/M}$**, c) metallacyclic complex (**M**) d) **$TS^2_{M/H\text{-}Q}$** e) hydroxo quinone complex (**H-Q**), f) **$TS^3_{H\text{-}Q/GD}$** and g) geminal diolate complex (**GD**). Here, the -CH_2- bridge of the **BPM** ligand stands up in **O_2-P**, in contrast to the structures with a down-standing bridge in Figure S8.

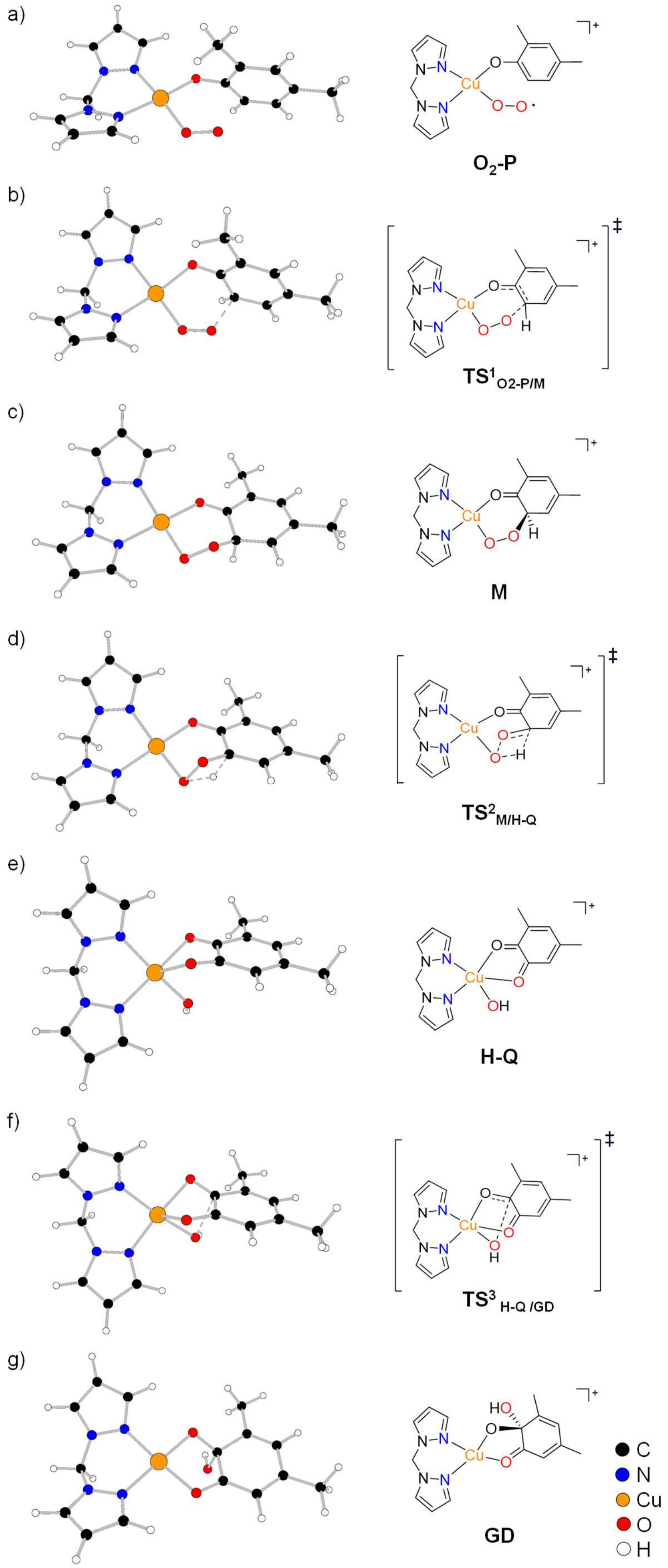

Figure S8. Intermediates and transition states of the mononuclear pathway of the **CuBPM** system including a) dioxygen phenolate complex (**O_2-P**) b) **$TS^1_{O2-P/M}$**, c) metallacyclic complex (**M**) d) **$TS^2_{M/H-Q}$** e) hydroxo quinone complex (**H-Q**), f) **$TS^3_{H-Q/GD}$** and g) geminal diolate complex (**GD**). Here, the -CH_2- bridge of the **BPM** ligand stands down in **O_2-P**, in contrast to the structures with an up-standing bridge in Figure S7.

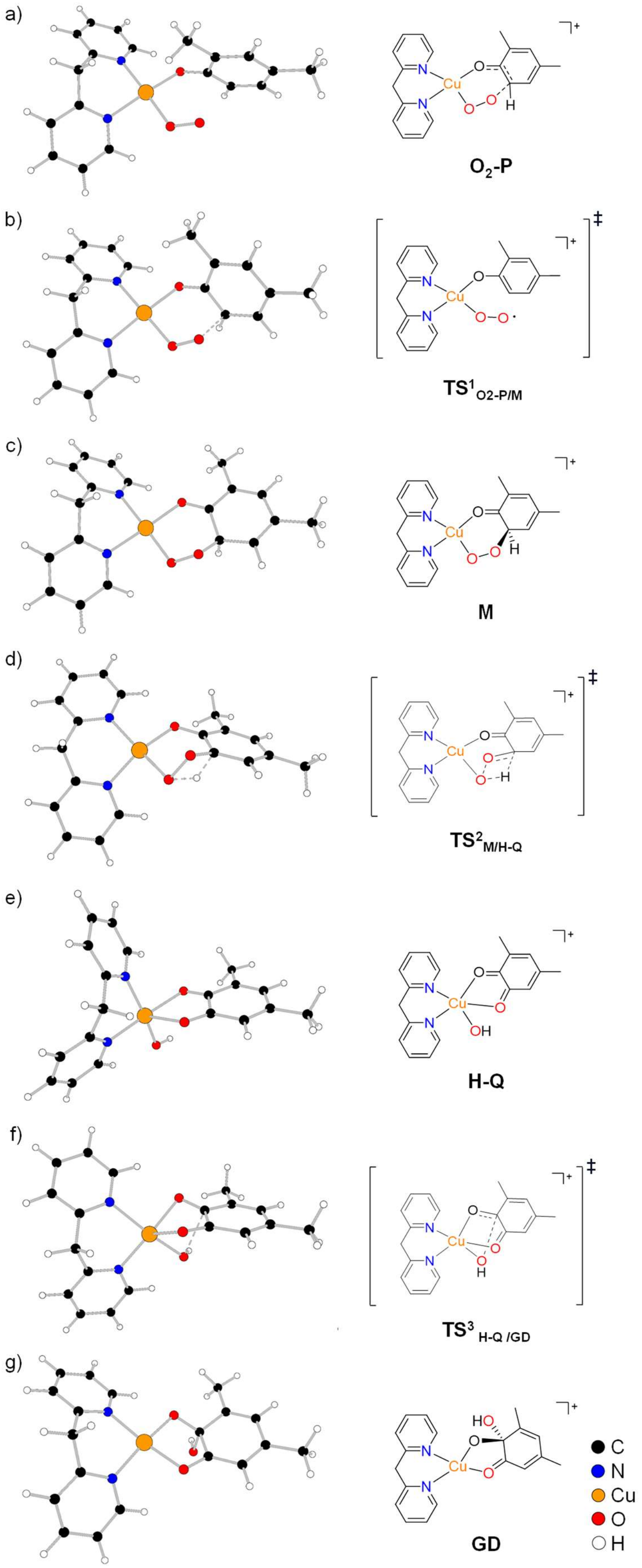

Figure S9. Intermediates and transition states of the mononuclear pathway of the **CuDPM** system including a) dioxygen phenolate complex (**O_2-P**) b) **$TS^1_{O2-P/M}$**, c) metallacyclic complex (**M**) d) **$TS^2_{M/H-Q}$** e) hydroxo quinone complex (**H-Q**), f) **$TS^3_{H-Q/GD}$** and g) geminal diolate complex (**GD**). Here, the -CH_2- bridge of the **DPM** ligand stands up in **O_2-P**, in contrast to the structures with a down-standing bridge in Figure S10.

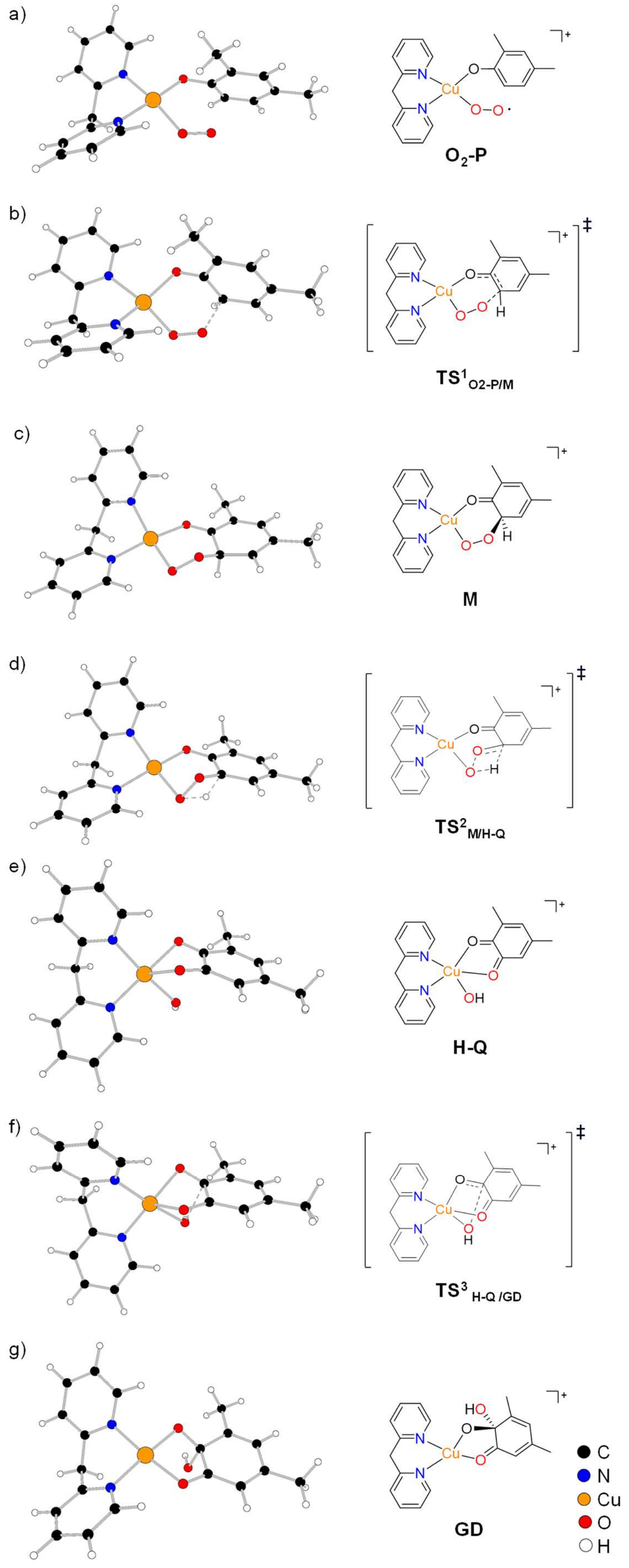

Figure S10. Intermediates and transition states of the mononuclear pathway of the **CuDPM** system including a) dioxygen phenolate complex (**O_2-P**) b) **$TS^1_{O2-P/M}$**, c) metallacyclic complex (**M**) d) **$TS^2_{M/H-Q}$** e) hydroxo quinone complex (**H-Q**), f) **$TS^3_{H-Q/GD}$** and g) geminal diolate complex (**GD**). Here, the -CH_2- bridge of the **DPM** ligand stands down in **O_2-P**, in contrast to the structures with an up-standing bridge in Figure S9.

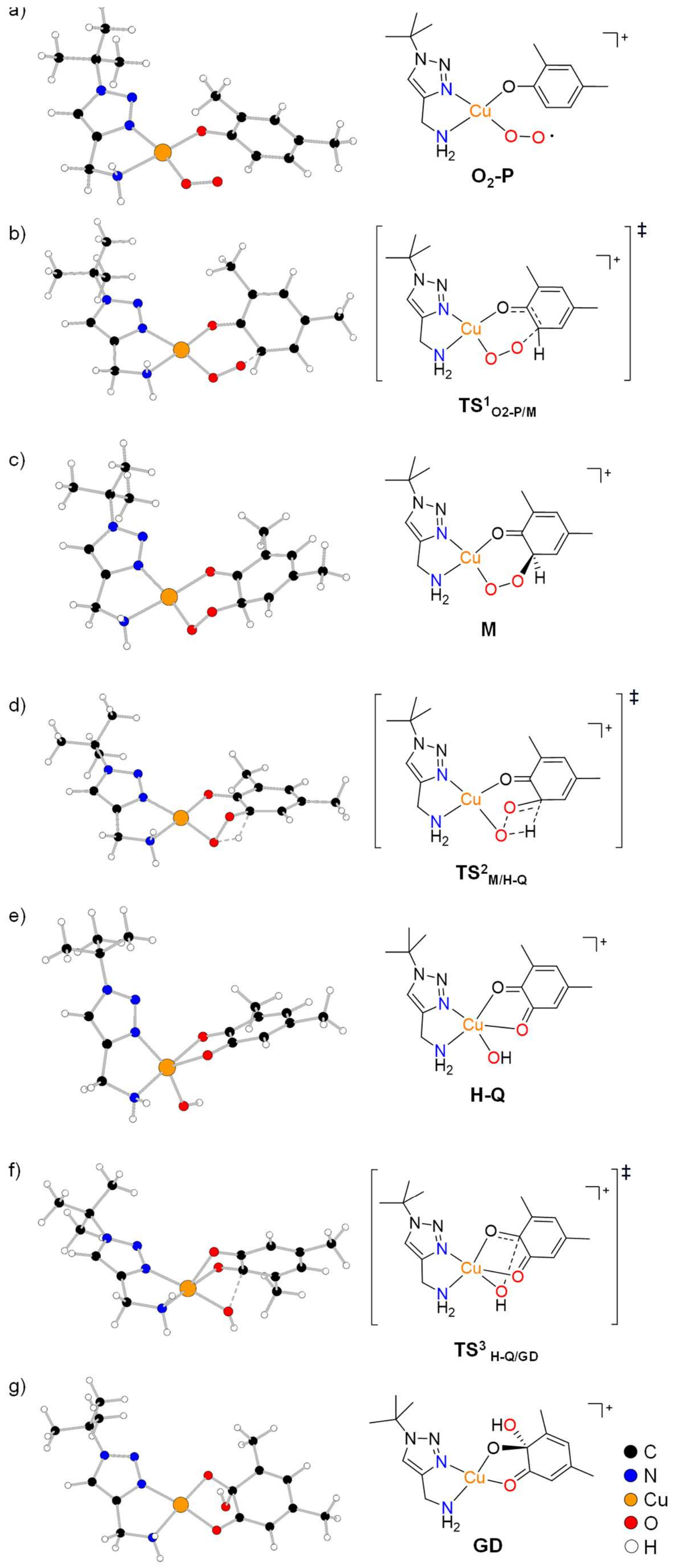

Figure S11. Intermediates and transition states of the mononuclear pathway of the **CuTTA** system including a) dioxygen phenolate complex (**O_2-P**) b) **$TS^1_{O2\text{-}P/M}$**, c) metallacyclic complex (**M**) d) **$TS^2_{M/H\text{-}Q}$** e) hydroxo quinone complex (**H-Q**), f) **$TS^3_{H\text{-}Q/GD}$** and g) geminal diolate complex (**GD**). Here, the amine donor of the **TTA** ligand coordinates *trans* to dioxygen ligand in **O_2-P**.

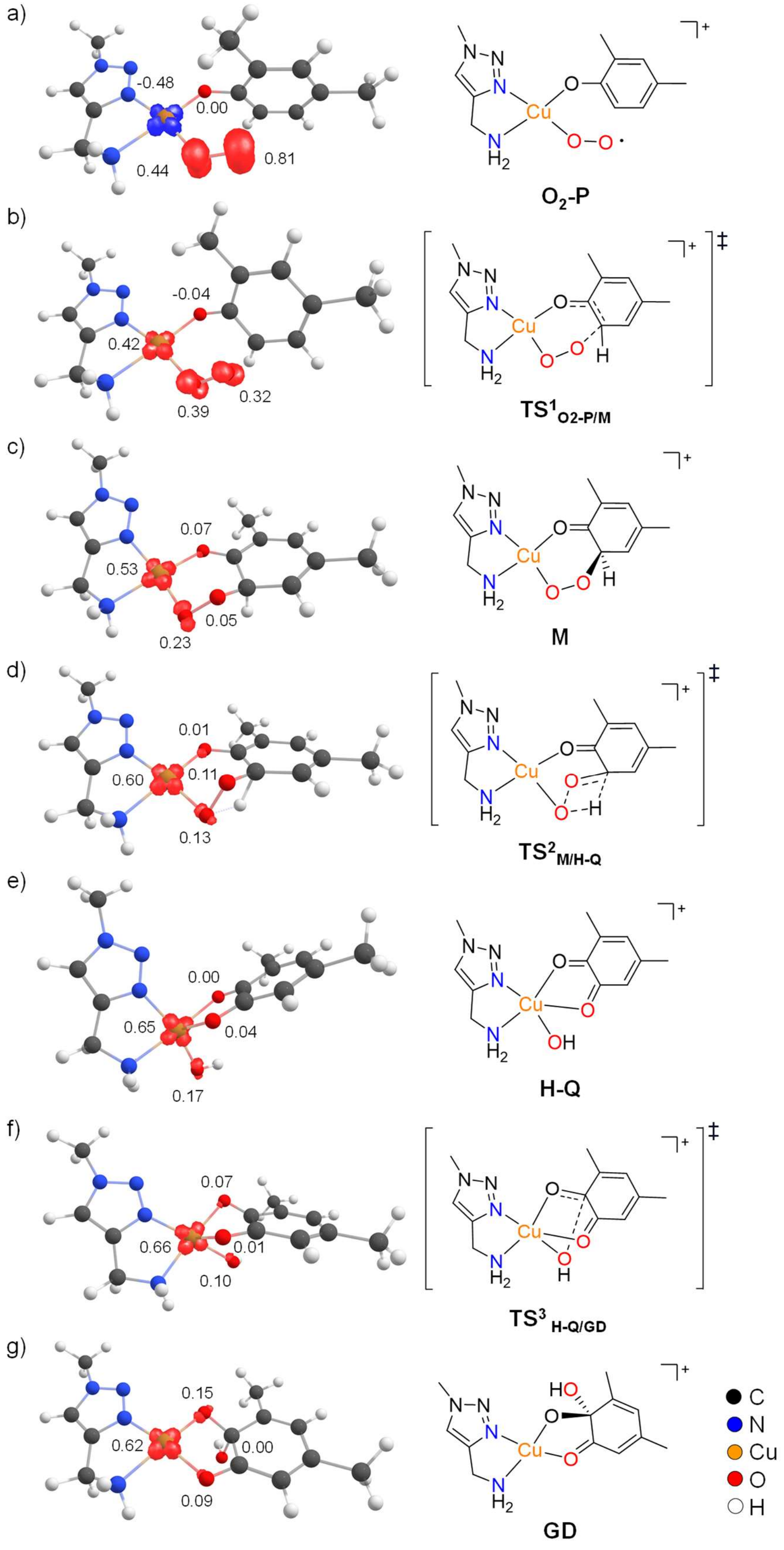

Figure S12. Spin densitities of the intermediates and transition states of the mononuclear pathway of the **CuMTA** including a) dioxygen phenolate complex (**O_2-P**) b) **$TS^1_{O2\text{-}P/M}$**, c) metallacyclic complex (**M**) d) **$TS^2_{M/H\text{-}Q}$** e) hydroxo quinone complex (**H-Q**), f) **$TS^3_{H\text{-}Q/GD}$** and g) geminal diolate complex (**GD**) including the Mulliken spin populations for the copper center and the three oxygen atoms. Red color represents alpha electron density and blue color represents the beta electron density.

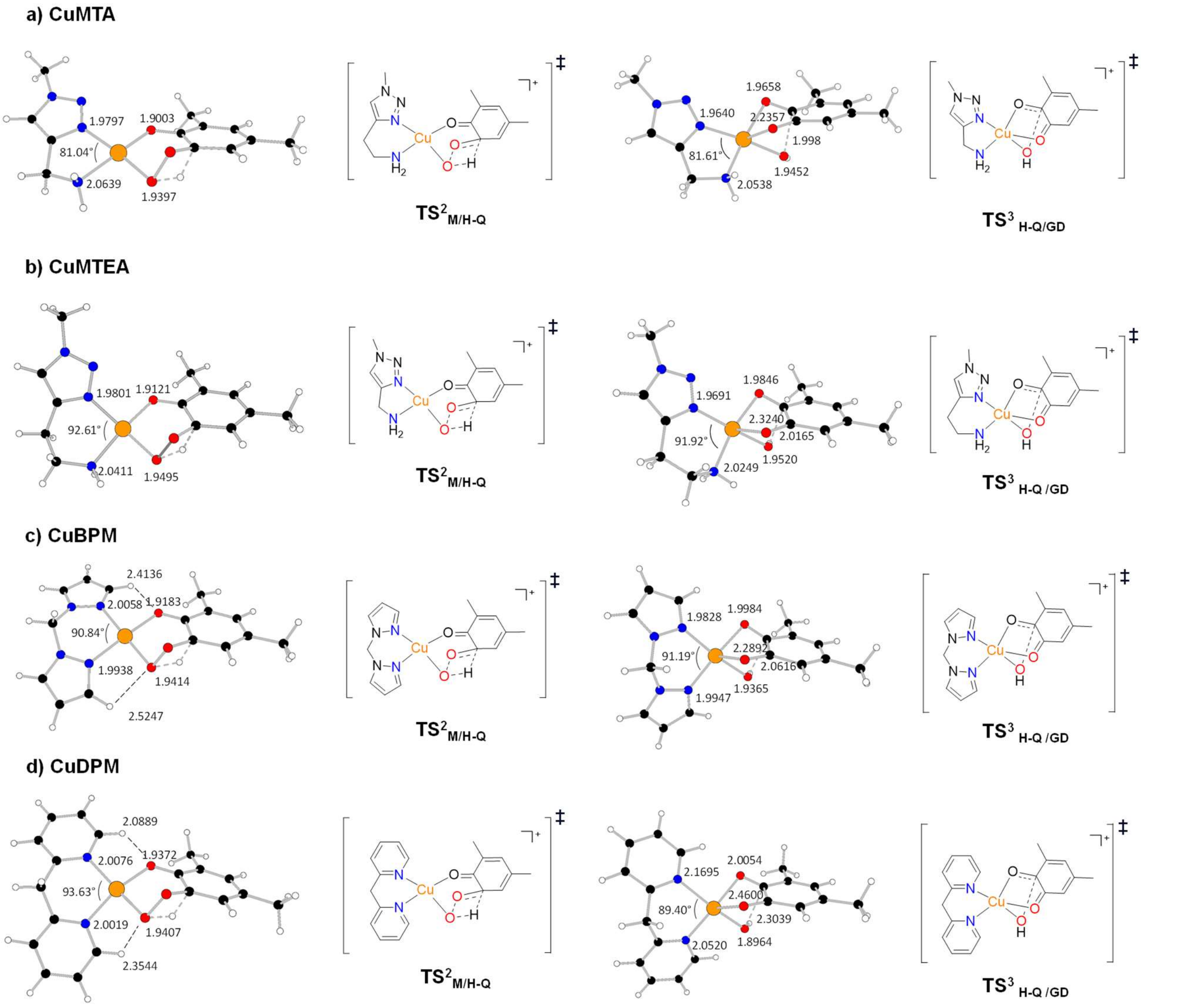

Figure S13. Comparison of the structures of **TS$^2_{M/H-Q}$** and **TS$^3_{H-Q/GD}$** transition states of the presented mononuclear pathway of the **CuL** systems (**L** = **MTA**, **MTEA**, **BPM**, **DPM**).

4. NMR spectrometry

The NMR spectrum was recorded at 300 K by use of a Bruker AVANCE III HD Pulse Fourier Transform spectrometer operating at frequency of 400.13 MHz (1H NMR) with TMS as internal standard. The catalytic activity of [Cu(**TTEA**)(NCMe)]PF_6 was investigated using a 500 µM solution of the copper(I) complex with 50 eq. of 2,4-di-tert-butylphenol and 100 eq. of triethylamine (BULKOWSKI-RÉGLIER conditions).[19] After stirring the solution under $^{18}O_2$ atmosphere for 5 h, the reaction was stopped by removing the solvent under vacuum.

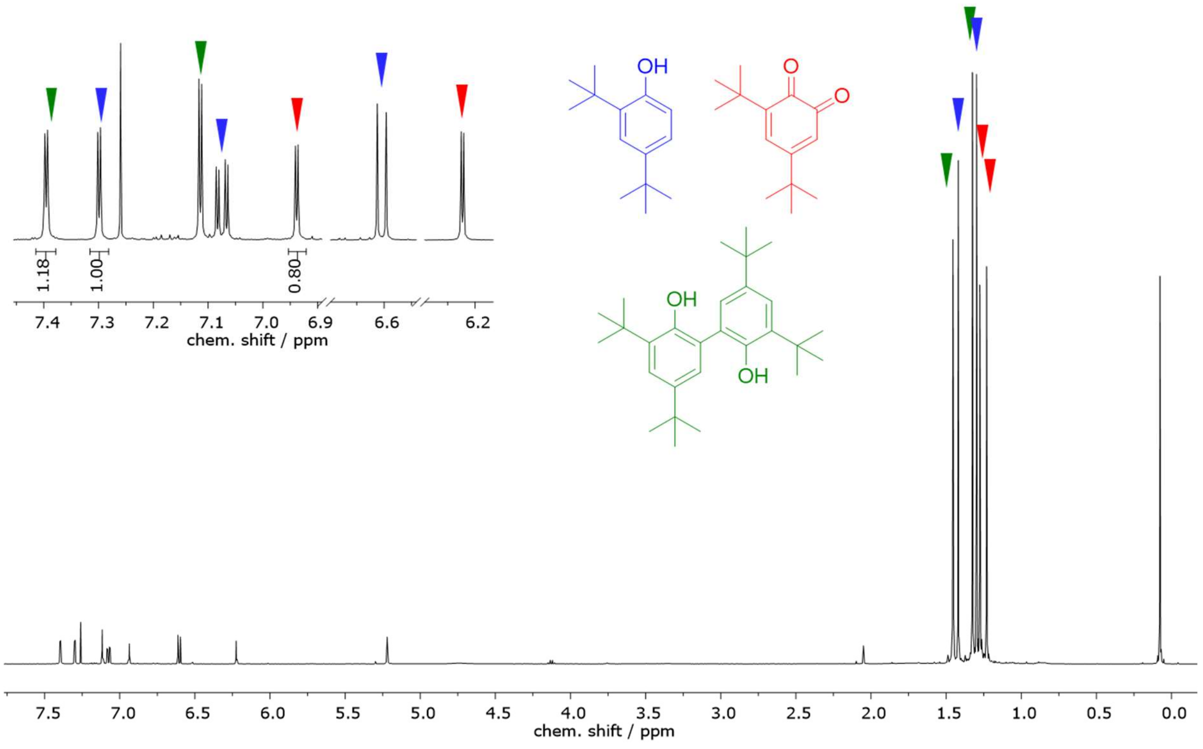

Figure S14. ^{1}H-NMR spectrum for the conversion of DTBP-H under $^{18}O_2$ atmosphere with [Cu(**TTEA**)(NCMe)]PF_6 as catalyst. The NMR spectrum was measured in deuterated chloroform.

5. Mass spectrometry

Mass spectrometric experiments were conducted on a triple quadrupole (Quattro II, Waters Co./Micromass Inc., Manchester, UK) equipped with an electrospray ionisation source (ESI, Z-spray). MS experiments were conducted under the following conditions: samples were prepared with the same procedure that was applied for the preparation of the NMR solutions but at 5 µM relative to DTBP-H in dry dichloromethane (LC grade, Chromasolv+, Honeywell Inc.) and analysed at 75 µL·min^{-1} flow rate using a syringe pump.

Capillary voltage was set at 4.5 kV, cone voltage at 20 V and extractor cone at 5 V. The source temperature was set at 100°C and desolvation heater at 130°C. Quadrupoles were set at unit resolution and argon (Alphagaz 2, Air Liquide S.A.) was used as collision gas in MS/MS experiments at $P=2.2\times10^{-3}$ mbar in the collision chamber (uncorrected Pirani gauge reading).

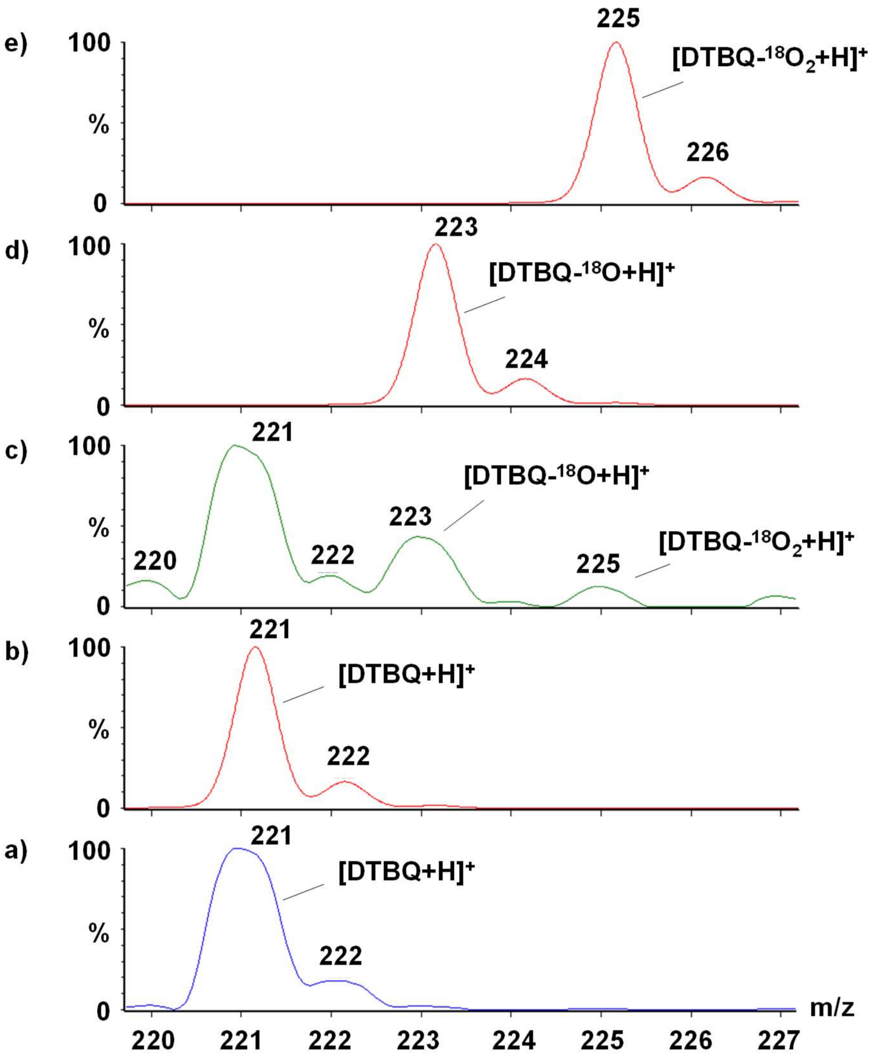

Figure S15. ESI-MS spectra of: a) commercial DTBQ (*m/z* 221) and b) its isotopic model; c) DTBQ obtained from the catalytic conversion of DTBP-H with $^{18}O_2$ (DTBQ-^{18}O at *m/z* 223 and DTBQ-$^{18}O_2$ at *m/z* 225); and d,e) isotopic models.

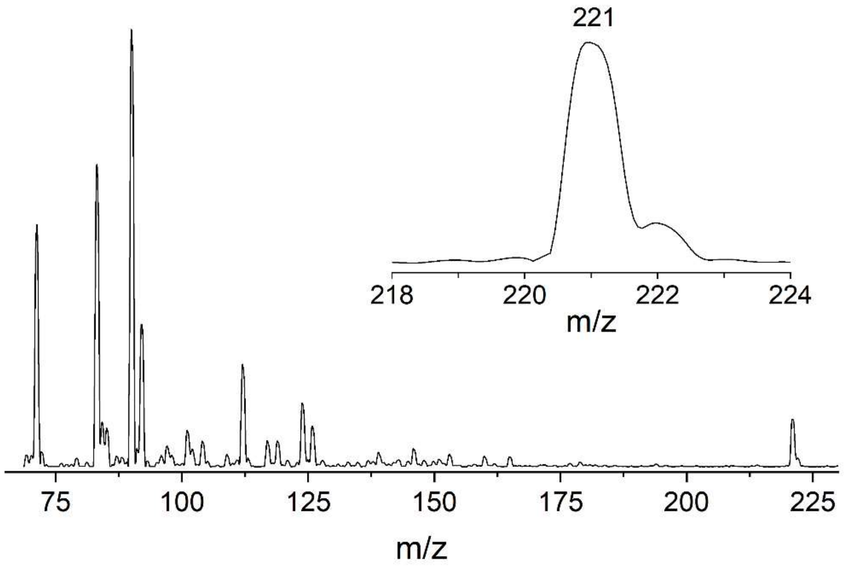

Figure S16. ESI-MS spectrum of commercially obtained DTBQ with enlarged peak for [M+H]$^+$ at *m/z* 221.

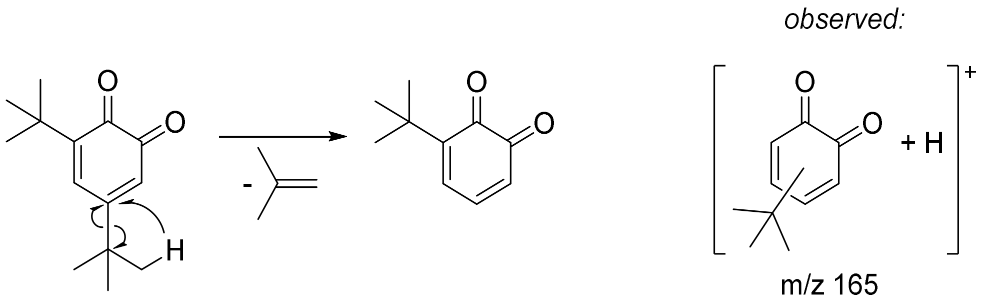

Scheme S1. Proposed reaction pathway for the formation of *tert*-butyl-quinone exemplified for elimination of the *tert*-butyl residue in 5-position.

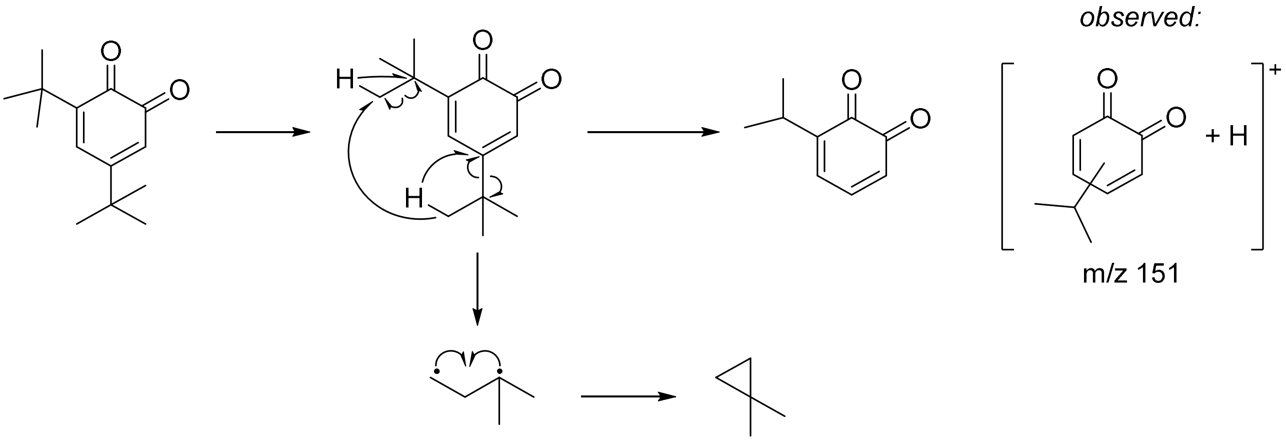

Scheme S2. Proposed reaction pathway for the formation of isoproyl-quinone exemplified for elimination of the *tert*-butyl residue in 5-position.

6. References

[1] F. Neese, *WIREs Comput. Mol. Sci.* **2018**, *8*, e1327.

[2] J. P. Perdew, K. Burke, M. Ernzerhof, *Phys. Rev. Lett.* **1996**, *77*, 3865.

[3] a) A. Hellweg, C. Hättig, S. Höfener, W. Klopper, *Theor. Chem. Acc.* **2007**, *117*, 587; b) F. Weigend, *Phys. Chem. Chem. Phys.* **2006**, *8*, 1057.

[4] S. Grimme, J. Antony, S. Ehrlich, H. Krieg, *J. Chem. Phys.* **2010**, *132*, 154104.

[5] S. Grimme, S. Ehrlich, L. Goerigk, *J. Comput. Chem.* **2011**, *32*, 1456.

[6] a) K. Eichkorn, O. Treutler, H. Öhm, M. Häser, R. Ahlrichs, *Chem. Phys. Lett.* **1995**, *240*, 283; b) K. Eichkorn, F. Weigend, O. Treutler, R. Ahlrichs, *Theor. Chem. Acc.* **1997**, *97*, 119; c) F. Neese, *J. Comput. Chem.* **2003**, *24*, 1740; d) F. Neese, F. Wennmohs, A. Hansen, U. Becker, *Chem. Phys.* **2009**, *356*, 98.

[7] V. Barone, M. Cossi, *J. Phys. Chem. A* **1998**, *102*, 1995.

[8] A. Koch, T. A. Engesser, C. Näther, F. Tuczek, *ChemCatChem* **2024**, *16*, e202301316.

[9] a) B. Herzigkeit, B. M. Flöser, N. E. Meißner, T. A. Engesser, F. Tuczek, *ChemCatChem* **2018**, *10*, 5402; b) A. Koch, T. A. Engesser, F. Tuczek, *Organometallics* **2023**, *42*, 1774.

[10] a) J. N. Hamann, R. Schneider, F. and Tuczek, *J. Coord. Chem.* **2015**, *68*, 3259; b) F. Wendt, C. Näther, F. Tuczek, *J. Biol. Inorg. Chem.* **2016**, *21*, 777.

[11] M. Rolff, J. Schottenheim, G. Peters, F. Tuczek, *Angew. Chem. Int. Ed.* **2010**, *49*, 6438.

[12] a) H. Jónsson, G. Mills, K. W. Jacobsen in *Classical and Quantum Dynamics in Condensed Phase Simulations*. Edited by B. J. Berne, G. Ciccotti, and D. F. Coker, World Scientific, **1998**, S. 385–404; b) V. Ásgeirsson, B. O. Birgisson, R. Bjornsson, U. Becker, F. Neese, C. Riplinger, H. Jónsson, *J. Chem. Theory Comput.* **2021**, *17*, 4929.

[13] a) J. P. Perdew, *Phys. Rev. B* **1986**, *33*, 8822; b) A. D. Becke, *Phys. Rev. A* **1988**, *38*, 3098.

[14] F. Weigend, R. Ahlrichs, *Phys. Chem. Chem. Phys.* **2005**, *7*, 3297.

[15] P. E. M. Siegbahn, *J. Biol. Inorg. Chem.* **2003**, *8*, 577.

[16] K. P. Jensen, B. O. Roos, U. Ryde, *J. Chem. Phys.* **2007**, *126*, 14103.

[17] M. M. Hagemann, E. D. Hedegård, *Chem. Eur. J.* **2023**, *29*, e202202379.

[18] B. Herzigkeit, R. Jurgeleit, B. M. Flöser, N. E. Meißner, T. A. Engesser, C. Näther, F. Tuczek, *Eur. J. Inorg. Chem.* **2019**, *2019*, 2258.

[19] a) M. Réglier, C. Jorand, B. Waegell, *J. Chem. Soc., Chem. Commun.* **1990**, 1752; b) J. E. Bulkowski, *US-Patent 4.545.937,* **1985**.
