# Supplementary figures and images for: A Mononuclear Scenario for the Copper‐Catalyzed Monooxygenation of Phenolic Substrates
Source: Chemistry. 2026 Mar 24;32(27):e03505. doi: 10.1002/chem.202503505 (PMC13380385; doi:10.1002/chem.202503505)

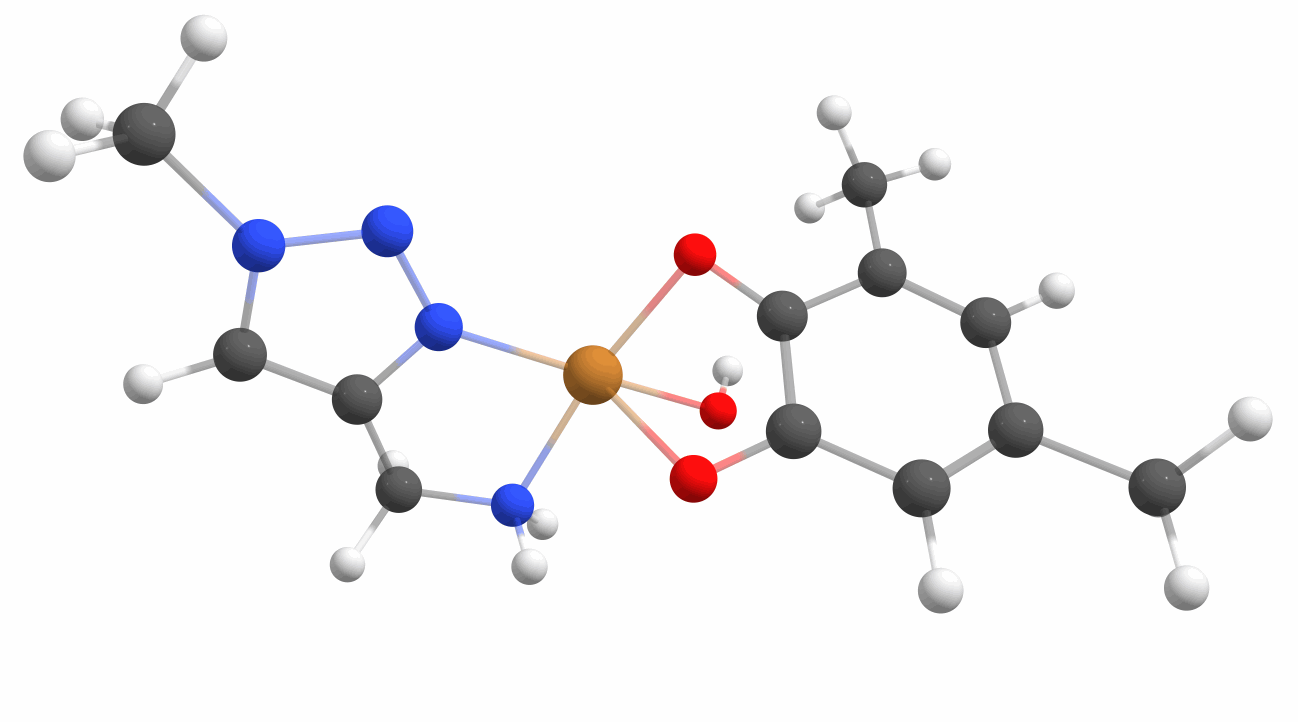

Supplement: Supplementary file 2 — Supporting File 2: chem70898‐sup‐0002‐SuppMat.zip. [file CHEM-32-e03505-s001.zip › TS3_HQ_GD.gif]

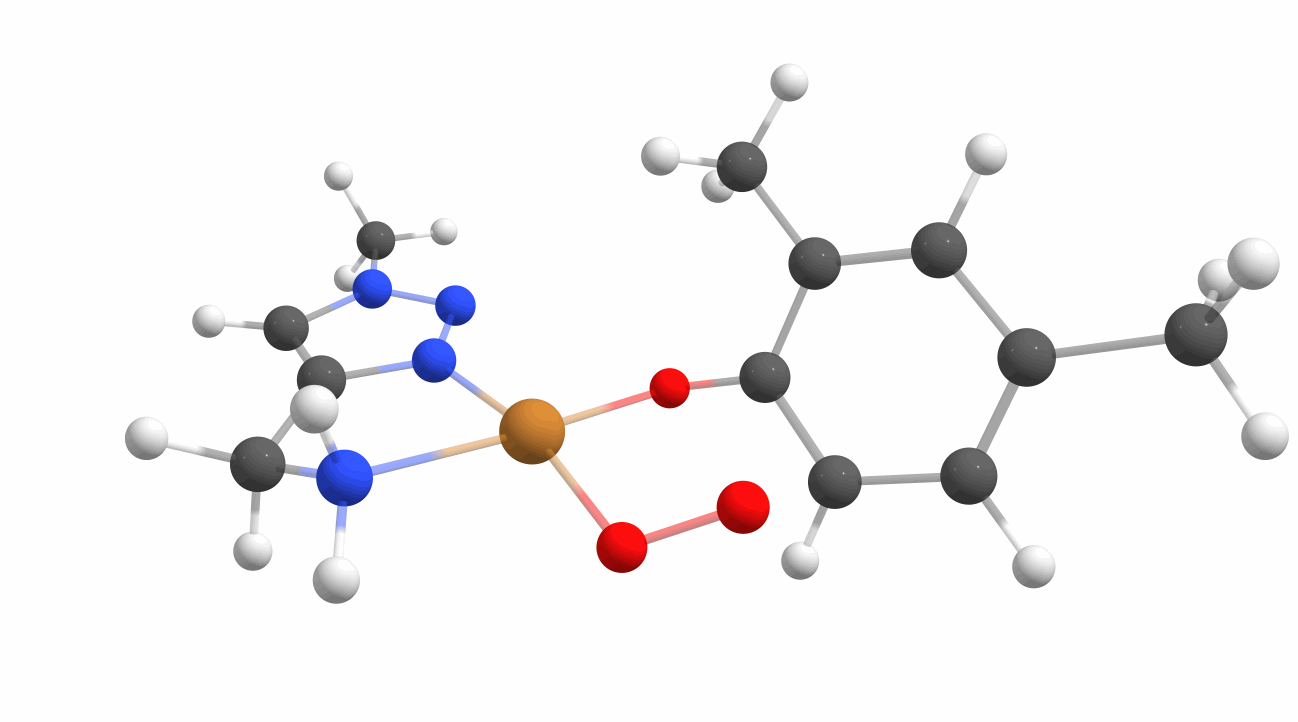

Supplement: Supplementary file 2 — Supporting File 2: chem70898‐sup‐0002‐SuppMat.zip. [file CHEM-32-e03505-s001.zip › TS1_S-P_M.gif]

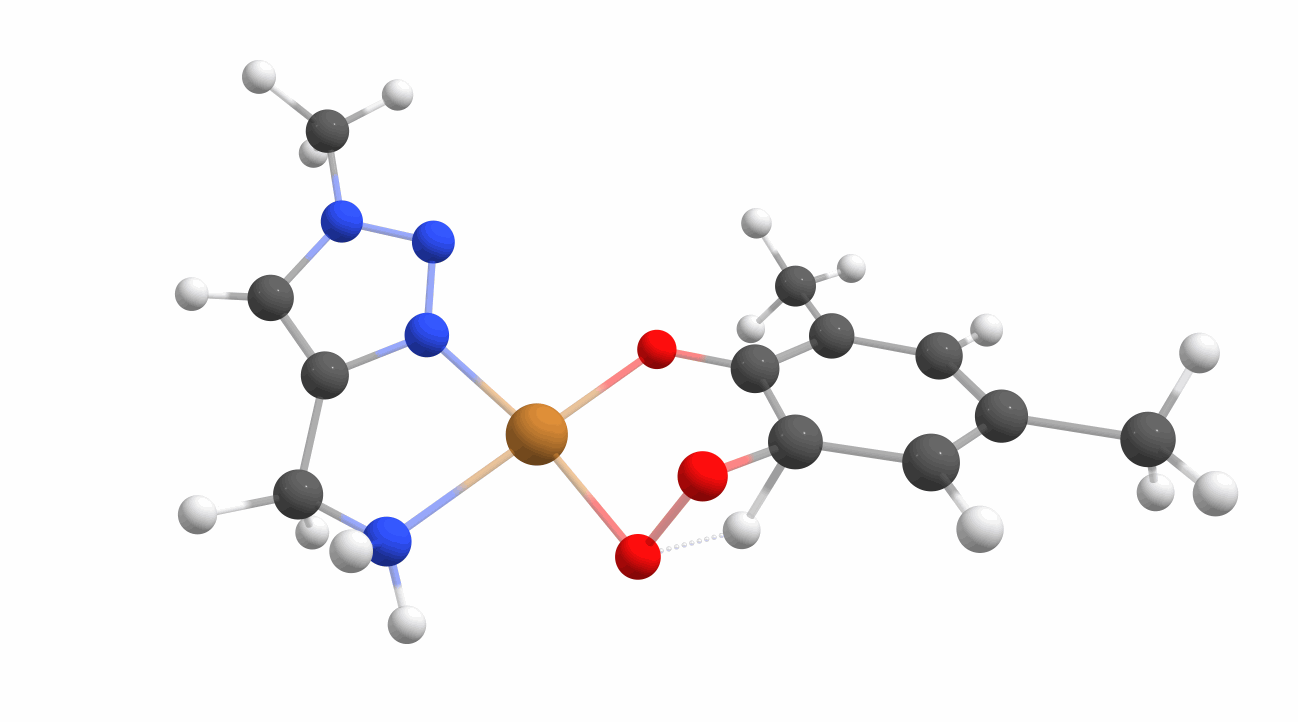

Supplement: Supplementary file 2 — Supporting File 2: chem70898‐sup‐0002‐SuppMat.zip. [file CHEM-32-e03505-s001.zip › TS2_M_HQ.gif]
